# Resistance to bacteriophage incurs a cost to virulence in drug-resistant Acinetobacter baumannii

**DOI:** 10.1099/jmm.0.001829

**Published:** 2024-05-14

**Authors:** Robyn Manley, Christian Fitch, Vanessa Francis, Isaac Temperton, Dann Turner, Julie Fletcher, Mitchelmore Phil, Steve Michell, Ben Temperton

**Affiliations:** 1 University of Exeter, Health and Life Sciences, Streatham Campus, Exeter, EX4 4QD, UK; 2 School of Applied Sciences, College of Health, Science and Society, University of the West of England, Bristol, Frenchay Campus, Coldharbour Lane, Bristol, BS16 1QY, UK; 3 University of Exeter, College of Medicine and Health, Department of Respiratory Medicine, Royal Devon & Exeter Hospital, Barrack Road, Exeter, EX2 5DW, UK

**Keywords:** *Acinetobacter baumannii*, *Galleria mellonella*, lytic, phage resistance, virulence

## Abstract

**Introduction*****.** Acinetobacter baumannii* is a critical priority pathogen for novel antimicrobials (World Health Organization) because of the rise in nosocomial infections and its ability to evolve resistance to last resort antibiotics. *A. baumannii* is thus a priority target for phage therapeutics. Two strains of a novel, virulent bacteriophage (LemonAid and Tonic) able to infect carbapenem-resistant *A. baumannii* (strain NCTC 13420), were isolated from environmental water samples collected through a citizen science programme.

**Gap statement.** Phage-host coevolution can lead to emergence of host resistance, with a concomitant reduction in the virulence of host bacteria; a potential benefit to phage therapy applications.

**Methodology.***In vitro* and *in vivo* assays, genomics and microscopy techniques were used to characterize the phages; determine mechanisms and impact of phage resistance on host virulence, and the efficacy of the phages against *A. baumannii*.

**Results.***A. baumannii* developed resistance to both viruses, LemonAid and Tonic. Resistance came at a cost to virulence, with the resistant variants causing significantly reduced mortality in a *Galleria mellonella* larval *in vivo* model. A replicated 8 bp insertion increased in frequency (~40 % higher frequency than in the wild-type) within phage-resistant *A. baumannii* mutants, putatively resulting in early truncation of a protein of unknown function. Evidence from comparative genomics and an adsorption assay suggests this protein acts as a novel phage receptor site in *A. baumannii*. We find no evidence linking resistance to changes in capsule structure, a known virulence factor. LemonAid efficiently suppressed growth of *A. baumanni in vitro* across a wide range of titres. However, *in vivo*, while survival of *A. baumannii* infected larvae significantly increased with both remedial and prophylactic treatment with LemonAid (10^7^ p.f.u. ml^–1^), the effect was weak and not sufficient to save larvae from morbidity and mortality.

**Conclusion.** While LemonAid and Tonic did not prove effective as a treatment in a *Galleria* larvae model, there is potential to harness their ability to attenuate virulence in drug-resistant *A. baumannii*.

## Data Summary

All sequence data of bacteria variants and phages and their assemblies have been added to GenBank (accession for LemonAid: OR608380, accession for Tonic: OR636104, SRAs for all data: PRJNA809500). Growth curve data, survival and melanization data from the *Galleria mellonella* experiments and data from the absorption assay, can be found on Dryad (doi: 10.5061/dryad.4j0zpc8jt).

## Introduction

The rise in number of antimicrobial-resistant (AMR) bacteria is a significant threat to human health [1]. The World Health Organization lists *Acinetobacter baumannii* as a critical priority pathogen for drug development [2]. This capsulated, Gram-negative, opportunistic bacteria is recognized as a global threat in clinical settings because of its rapid emergence of resistance to current antibiotics, including carbapenems [3]. Carbapenems are the last line of defence against multidrug-resistant (MDR) *A. baumannii* infections before resorting to more toxic antibiotics, such as polymyxins, that can incur serious side effects (reviewed in [4]). Carbapenem-resistant *A. baumannii* is estimated to be associated with over 400 000 deaths worldwide in 2019 [1]. *A. baumannii* is capable of surviving for prolonged periods on dry surfaces [5] such as hospital tools, increasing its potential for nosocomial spread. Patients in intensive care are vulnerable to infection by *A. baumannii* via invasive tools, leading to life-threatening infections such as pneumonia, meningitis, urinary tract, and blood and soft tissue infections [6,7].

The cost of screening and obtaining regulatory approval of new antibiotic compounds has stimulated the search for alternative treatments [8]. Bacteriophage (phage) therapy, the clinical use of viruses that kill pathogenic bacteria, has seen a resurgence of interest in Western countries with phage banks being developed across the world (https://www.bacteriophage.news/database/) and an increase in applications for compassionate phage use, i.e. when a patient has exhausted standard-of-care treatments [9]. A recent systematic review [10] reports that 2241 patients were treated with phage therapy between 2000 and 2021, across a broad range of pathologies, with clinical improvement seen in 78 % of cases. At the time of writing, there are six active clinical trials for phage treatments listed on https://clinicaltrials.gov and a nationwide personalized phage clinical trial underway in Australia (https://www.phageaustralia.org/) [11]. To date there are three published cases of compassionate use of phage therapy in the UK [12,14].

The concerning rise in case numbers of *A. baumannii* infections and its ability to evade antibiotics makes it a priority target for phage therapy. Over 130 phages that infect *A. baumannii* have been isolated [15] and the effectiveness of phages against * A. baumannii* infection has been demonstrated in both mouse [16,18], and human plasma models of infection [19]. Crucially, there have been a number of human patients with *A. baumannii* infections where phages, in combination with antibiotics, improved clinical outcomes [20,24].

A potential barrier to phage therapy is the ability of bacteria to develop resistance during treatment. Virulent phage infections apply a strong selective pressure on host bacteria to evolve defence mechanisms [25,26]. For example, bacteria can evolve changes to phage receptor proteins on the cell surface that can reduce or prevent the phage from adsorbing and entering the cell [26]. Such surface changes can come with trade-offs, often leading to reduced survival or virulence of the host bacteria, or increased susceptibility to antibiotics [27,28]. The capsule of *A. baumannii* is a key virulence factor [29] and the emergence of phage resistance in *A. baumannii* has been associated with genetic mutations in capsule genes, which lead to changes in colony morphology, reduced polysaccharide production and reduced virulence [18,30,32].

One model for studying phage resistance-virulence trade-offs, and to test the efficacy of phage as a potential therapy, is the wax moth larvae, *Galleria mellonella. G. mellonella* are considered a useful and reliable model for studying multi-drug-resistant (MDR) *A. baumannii* pathogenesis [33]. They provide a low-cost, *in vivo* model for evaluating bacteria–phage interactions in the presence of an immune system, prior to use of mammalian models and thus, offer an important step-up from *in vitro* experiments. *G. mellonella* has a complex innate immune system, with similarity to mammalian systems [34] and can be kept at 37 °C during experiments to approximate conditions in human infections. *G. mellonella* have been used to assess changes in virulence of phage-resistant *A. baumannii* to further understand mechanisms of resistance [18,32]. Several studies have used *G. mellonella* to test the efficacy of virulent phage against MDR strains of *A. baumannii* and observed improved larvae survival with phage treatment [35,37]. Further, two studies using both *G. mellonella* and mouse models found comparable survival effects [16,17]. *G. mellonella* have also been used to test the efficacy of phage–antibiotic combination therapies, with positive results recorded against MDR *A. baumannii* [19,36, 37].

Here, we describe two new phages of the same species with myovirus morphology, LemonAid and Tonic, isolated against a clinical carbapenem-resistant *A. baumannii* strain (NCTC 13420) from a UK hospital outbreak in the early 2000s [38]. We identify putative mechanisms of phage resistance using comparative genomics and microscopy, and demonstrate a fitness cost to this resistance that translated to reduced virulence *in vivo*. Further, therapeutic potential was determined *in vitro* and in a *G. mellonella* model.

## Methods

### The Citizen Phage Library (https://www.citizenphage.com) methodology for phage hunting

*Acinetobacter* phage LemonAid was isolated from a water sample provided by a citizen scientist from the River Lemon in Devon, downstream of a wastewater storm overflow (50.525 N, 3.626 W). Tonic was isolated from raw sewage samples collected by the Environment Agency during routine monitoring of SARS-CoV-2 during the COVID-19 pandemic. Upon collection, samples were transferred to the lab and filtered through 0.22 µM pore syringefilters (polyethersulfone membrane, Merck, Millipore) to remove bacterial cells and particulate debris. Filtrates were enriched for *A. baumannii* phage using a protocol adapted from Olsen *et al*. [39]. *A. baumannii* strain NCTC 13420 (henceforth referred to as *A. baumannii*) was grown overnight in Luria–Bertani (LB) broth, containing CaCl_2_ and MgCl_2_ at a final concentration of 10 mM (henceforward defined as LB/Ca/Mg, unless otherwise stated), at 37 °C with shaking at 200 r.p.m. A 1 ml volume of filtrate was added to a deep 96-well plate (VWR, USA) and mixed with LB (final concentration x1), CaCl_2_/MgCl_2_ solution (final concentration of 10 mM) and 5 % v/v of overnight host culture, in a final volume of 1.5 ml. Covered plates were incubated overnight at 37 °C degrees on an orbital shaker at 200 r.p.m. The following day, 200 µl of each sample was filtered through a 0.45 µm pore filter MultiScreenHTS HV sterile filter plate (PVDF membrane, Millipore, Merck) placed on top of a 96-well microtitre plate (Grenier Bio-One, Austria) by centrifugation at 900 ***× g*** for 4 min. Then, 5 µl of filtrate containing enriched phages was used in a second round of infection in LB/Ca/Mg with fresh overnight culture of *A. baumannii*, incubated at 37 °C with shaking. Host cells were removed the following day using a 0.45 µm pore filter plate and the filtrate was screened for enriched phages infecting *A. baumannii* using a plaque assay. Plaques were subsequently purified through three rounds of dilution-to-extinction plaque assays (method S1). Purified phages were propagated overnight in 2×20 ml cultures of exponentially growing *A. baumannii*, centrifuged for 30 min at 10 000 × ***g*** at 4 °C and syringe filtered through 0.22 µm pore filter. The filtrate was stored in glass amber bottles at 4 °C as master stocks for the Citizen Phage Library. Aliquots of the master stock were carried forward for transmission electron microscopy, DNA extractions and infection experiments, described below.

### Phage DNA extraction, sequencing, assembly, genome annotation and phylogeny

A 30 ml volume of phage filtrate was treated with DNase 1 (5 mg ml^–1^, Roche) and RNase A (10 mg ml^−1^, Invitrogen) for 30 min at 37 °C to remove non-encapsulated (e.g. free bacterial DNA) nucleic acids from the lysate. Polyethylene glycol 8000 (PEG) and NaCl were dissolved in the lysate to final concentrations of 10 % w/v and 1.12 M, respectively, and left overnight at 4 °C. Phages were concentrated by centrifugation at 10 000 × ***g*** for 10 min, the supernatant removed and the phage pellet re-suspended in 1 ml of SM buffer (100 mM NaCl, 50 mM Tris-HCL and 8 mM MgSO_4_-H_2_0). DNA was purified using Promega Wizard Genomic DNA Purification kit following the manufacturer’s instructions and quantified using a broad-range Qubit dsDNA Quantification Assay kit and quality checked using Genomic DNA Screen Tape analysis on the Agilent 4200 TapeStation system, as per the manufacturer’s instructions. DNA sequencing libraries were prepared using NEBNext Ultra II FS Library Preparation and run on the Illumina Novaseq by the Exeter Sequencing Service, to generate 2×150 bp paired end reads, that were filtered and trimmed using MultiQC (v.10.1) [40]

High-quality reads were assembled with Unicycler v.0.5.0 [41] and contigs >20 kb assessed with CheckV (v.0.8.1) [42] and DRAM-v (v.1.2) [43] to identify high-confidence viral contigs. Genomes were annotated using Pharokka v.1.3.0 [44] with default settings [45]. Genomes were analysed with Phage.ai [46] to predict if the life cycles were virulent or temperate. Whole-genome comparison of LemonAid and Tonic was performed with the progressiveMauve algorithm [47] in Geneious v.10.1 (https://www.geneious.com). Antimicrobial resistance genes and virulence factors were screened within the phage genomes during standard Pharokka annotation against the VFDB (a bacterial virulence factor database) [48] and CARD (an antimicrobial resistance database) [49] databases and PhageLeads [50]. Bacteriophage genomes exhibiting similarity to Tonic and LemonAid were identified using blastn searches and analysed with ViPTree [51] (https://www.genome.jp/viptree/), and these related phage genomes were analysed alongside our phage genomes using viridic [52] (https://rhea.icbm.uni-oldenburg.de/viridic/) to determine similarity to existing phage isolates.

### Prophage genetics

Two additional phage contigs were recovered at low coverage from the LemonAid assembly. To assess whether the LemonAid lysate represented a mixed sample of bacteriophages or if these contigs represented induced prophages, sequence reads were mapped against the NCTC 13420 genome: raw reads from the LemonAid lysate were recruited against the A. *baumannii* genome using minimap2, with mapped reads filtered using coverM (https://github.com/wwood/CoverM) to retain proper pairs with >98 % nucleotide identity across >100 bp read length. The region of the *A. baumannii* genome between 2 672 165 bp and 2 761 043 bp was shown to have high recruitment, indicating a potential induced prophage. This region was extracted with biopython and annotated with pharokka as described previously. Coverage of the *A. baumannii* genome was visualized in Geneious Prime and exported for final visualization in RStudio. To assess distribution of similar prophage clades represented by Fizzy and Cloudy, blastn was performed against a dataset of 420 complete *A. baumannii* genomes where putative prophage regions had been predicted using PhiSpy (Turner, unpublished data) (method S2).

### Transmission electron microscopy (TEM) imaging of phage

Phages were transferred onto electron microscopy (EM) grids (pioloform-coated 100 mesh, Agar Scientific) by floating the grids on droplets of virus suspension for 3 min, washed four times for 3 min on droplets of deionized water, before negative staining on droplets of 2 % w/v uranyl acetate for 3 min. Excess stain was removed using filter paper. After air-drying, samples were visualized using a JEOL JEM 1400 transmission electron microscope operated at 120 kV and images were taken with a digital camera (Gatan ES 1000W, Ametek).

### Phage-resistant cultures

After overnight incubation of *A. baumannii* with both LemonAid and Tonic, resulting cultures were turbid, suggesting strong bacterial growth and phage resistance. After streaking, a colony from each of these cultures was selected and determined by spot assay (method S1) to be resistant to LemonAid or Tonic, respectively, as well as cross resistance to both phage. These two phage-resistant variants (referred to as LemonAid resistant and Tonic resistant, or together as phage resistant, variants) were cryostored and used in growth curve assays.

### *In vitro* growth curve assays

*(a) Phage-bacteria dynamics:* A 96-well plate containing LB/Ca/Mg was inoculated with 2 µl of host cells (exponential phase, OD_600_ ~0.6 (optical density measured at 600 nm wavelength)) and 5 µl LemonAid or Tonic lysate in serial dilution from 10^9^ to 10^1^ p.f.u. ml^−1^, to a final volume of 100 µl per well. Negative controls containing LB/Ca/Mg only and LB/Ca/Mg with phage lysate only, and a positive control of phage-free host cells, were run on the same plate. Plates were incubated at 37 °C and 200 r.p.m. for 15 h, and optical density measurements were taken every 30 min on a microplate reader (Infinite 200 Pro, Tecan). All statistical analyses were carried out in R (v .4.1.2) and R studio (v.2021.09.2–382). Bacterial growth curves were plotted with ggplot (v.3.3.5) [53]. Virulence of LemonAid concentrations against *A. baumannii* was calculated from the growth curves as 1 – area under the curve (AUC) phage treatment/AUC (no phage) [54].

*(b) Fitness of phage-resistant variants:* Fitness of wild-type *A. baumannii* and phage-resistant variants were compared using growth curves as described above, using 98 µl of LB/Ca/Mg and 2 µl of exponential phase cultures in eight replicate wells per variant. The package growthcurver v.0.3.1 [55] was used to calculate growth rate (*r*), carrying capacity (*k*) and AUC to compare the phage-resistant variant to the wild-type *A. baumannii*. To test for statistical differences between the means of elements of growth, a Wilcoxon test from the package rstatix v.0.7.0 [56] was used, followed by the package coin v.1.4.2 [57] calling wilcox_effsize, to calculate effect sizes and confidence intervals by bootstrap (nboot=1000).

### Sequencing of wild-type and phage-resistant variants of *A. baumannii* NCTC 13240

Wild-type and phage-resistant variants were cultured for sequencing to identify the associated mutations as follows: Batch cultures of *A. baumannii* were prepared in 20 ml LB/Ca/Mg and incubated overnight, with and without LemonAid or Tonic. Cultures were centrifuged, supernatants removed and cell pellets were re-suspended in 5 ml of PSB ×1 to an OD_600_ of 1. DNA was extracted from 1 ml of culture using the Circulomics Nanobind high molecular weight genomic DNA extraction kit. Wild-type *A. baumannii* DNA was used for long-read (Oxford Nanopore, Rapid Sequencing kit RAD004) and short-read (Illumina, NEBNext Ultra FSII) sequencing (method S3). Hybrid assembly of short- and long-read sequence data was performed using Unicycler (v.0.5.0) [41] and the resulting wild-type reference genome was annotated using Prokka (v.1.14.6) [45]. Phage-resistant variants were sequenced with short-read sequencing only. Short-read data of wild-type and resistant genotypes were aligned to the wild-type reference genome with minimap2 (v.2.24) [58]. Single nucleotide polymorphism (SNP) calling and visualization was performed using three tools, Geneious v.10.1, bcftools mpileup [59] and Breseq [60], using consensus SNPs to reduce false positives. Unmapped short reads were assembled *de novo* using Shovill v.1.1.0 (https://github.com/tseemann/shovill) to determine if resistance was a product of gained genes or plasmids. Informatic tools were employed to find evidence for the function of a hypothetical protein with an insertion of interest (method S4).

### Adsorption assay of LemonAid against wild-type *A. baumannii* and LemonAid-resistant *A. baumannii*

Phage adsorption to *A. baumannii* wild-type and LemonAid-resistant genotype was assessed, adapting methods described by Alseth *et al*. [27], by monitoring phage titres over time during infections (at 0, 2, 4, 6 and 10 min), after inoculating each bacterial genotype in exponential phase (1×10^8^ c.f.u.) with LemonAid at 1×10^6^ p.f.u. (final m.o.i.=0.01). A bacteria-free control was sampled at 0 and 20 min. Assays were carried out in 1 ml reactions (three replicates for each time point) containing 500 µl 2×LB/Mg/Ca, incubated at 37 °C with shaking at 200 r.p.m. At each time point, samples were placed on ice and 200 µl of each sample was immediately transferred to a 0.45 µm pore filter plate and centrifuged to remove bacteria cells. After the time course was completed, the filtered samples were diluted and spotted onto lawns of *A. baumannii* wild-type. Plaques were counted after 24 h incubation at 37 °C.

### Scanning electron microscopy (SEM) and capsule staining to evaluate changes to bacterial cell surface structure in phage-resistant variants

Scanning electron microscopy was used to evaluate whether resistance was a function of alterations in cell-surface features. *A. baumannii* wild-type and phage-resistant cells were fixed in suspension in 2 % glutaraldehyde, 2 % paraformaldehyde in 0.1 M sodium cacodylate (pH 7.2) for 2 h at room temperature, washed three times with cacodylate buffer and post-fixed with 1 % osmium tetroxide in deionized water for 1 h. Cells were washed three times with deionized water, dehydrated through a graded ethanol series and filtered onto a 0.2 µm polycarbonate filter with gentle vacuum. Filters were treated with hexamethyldisilazane (Merck) for 3 min before air-drying. After sputter coating the sample with 10 nm gold/palladium (Q150T sputter coater, Quorum), samples were imaged with a Zeiss GeminiSEM 500 operated at 5 kV using an SE2 detector.

*A. baumannii* wild-type and phage-resistant variants were examined for the presence or absence of a capsule using capsule differential staining methods – Anthony’s capsule stain and Maneval’s staining method following [61]. The cells were examined at 100×using oil immersion phase contrast light microscopy.

### *In vivoG. mellonella* infection assays

Phage and bacteria inocula were prepared (method S5). Adapting methods in Champion *et al*.[62], larvae were divided into treatment groups, discarding any that were discoloured or appeared to be in poor health. A 250 µl Hamilton syringe was used for inoculations. Larvae were inoculated with 10 µl of inoculum in the first left proleg. Where two inoculations were necessary (i.e. when both bacteria and phage were delivered), the second inoculation was delivered into the first right proleg after a 30 min rest period (Fig. S1, available in the online version of this article). Inoculated larvae were stored on filter paper in petri dishes and replaced if they were injured or lost haemolymph from the injection site. Negative controls inoculated with 1×PBS and no-stab controls were observed alongside each experiment. For more details see method S6.

- To assess the virulence of phage-resistant variants larvae were inoculated with a dose of ~5×10^6^ WT *A*. *baumannii* and the LemonAid-resistant and Tonic-resistant *A. baumannii* variants (*N*=30 per treatment group), alongside negative controls as described above.
- To assess the efficacy of LemonAid against *A. baumannii* in *G. mellonella:* Firstly, larvae were inoculated with 10 µl of *A. baumannii* in 1 : 10 serial dilutions at a starting dose of 4×10^6^ c.f.u. (*N*=10 per treatment) to determine if infection of larvae with *A. baumannii* reduces survival in a dose-dependent manner, to obtain the best dose to use in the efficacy assay below and calculate the LD50. Secondly, to assess if LemonAid reduces mortality or melanization of larvae infected with *A. baumannii*, larvae were inoculated with *A. baumannii* only (positive control), LemonAid-only and PBS (negative controls), and remedial and prophylactic treatment of *A. baumannii* inoculated larvae with LemonAid (Table S1). LemonAid dose was 5×10^6^ p.f.u., thus a m.o.i. of ~1. An additional assay was run to evaluate remedial LemonAid treatment of larvae inoculated with a lower dose (LD50) of *A. baumannii* at 4×10^5^ c.f.u.

After inoculations, larvae were transferred to specially designed 3D printed plastic plates (Biosystems Technology) with wells to separate and contain individual larvae (Fig. S2a) and kept at 37 °C. Melanization and survival of larvae was evaluated every 2 h. Melanization was quantified using brightfield images of individual larvae analysed using the software IMPACT2AMR (https://github.com/ashsmith88/IMPACT2AMR_galleria_imaging). IMPACT2AMR uses machine learning to identify larvae within a boundary and quantifies pixel brightness (inversely proportional to melanization) within the larvae outline. Treatments were distributed evenly across the plates to randomize differences in light exposure across the plate that could otherwise affect melanization scores (Fig. S2a–c). Survival was determined as described previously [62].

For all *G. mellonella* data, the Survival package (v.3.5–5) [63] was used to produce Kaplan–Meier survival curves of the * G. mellonella* assays using the survfit function, and the survdiff function to test the difference between curves with a log-rank test. To determine if there were differences in melanization of larvae between treatment groups, lme4 (v.1.1–31) [64] was used to run linear models, modelling melanization as dependent on treatment, time (as a factor variable) and the interaction between treatment and time (melanization ~treatment*time). If applicable, plate number was included as a fixed effect. Residual plots were examined to determine goodness of fit.

### Endotoxin testing

LemonAid phage lysate was tested for endotoxin levels using the ToxinSensor chromogenic LAL endotoxin assay kit (GenScript, UK) following the manufacturer’s instructions (methods S7).

## Results

### LemonAid and Tonic morphology, genetics and phylogenetics

**Fig. 1.**
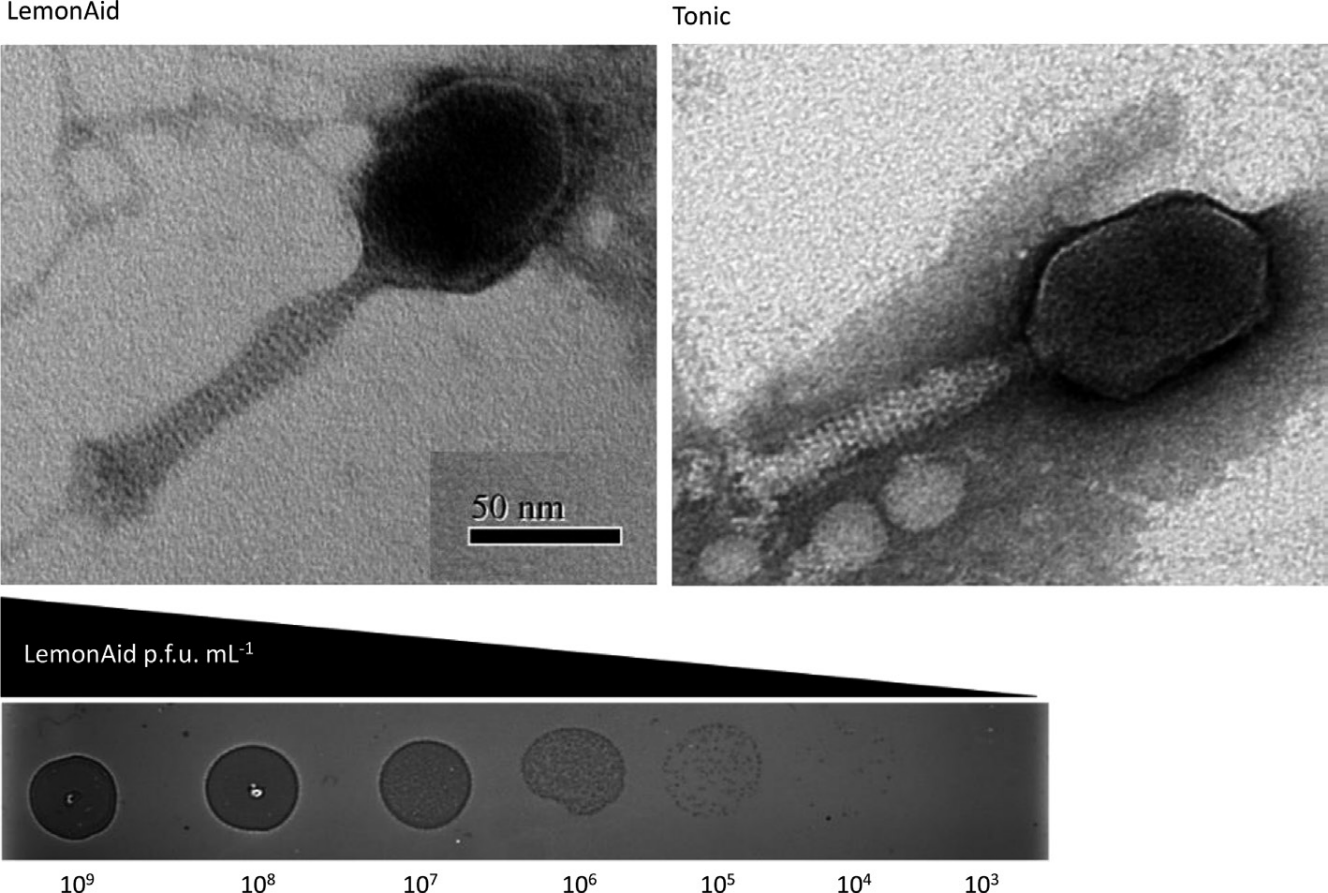
**(a**) Transmission electron microscope images of novel Myovirus’ LemonAid and Tonic, (**b**) LemonAid plaques on agar in dilution series (p.f.u.=plaque forming units).

TEM revealed that LemonAid and Tonic have a prolate head and contractile tail typical of a myovirus (Fig. 1a). LemonAid and Tonic infect *A. baumannii* NCTC 13420 forming small, clear plaques on LB agar (0.7 % w/v agar, mean diameter ~0.5 mm) (Fig. 1b).

Assembly of sequencing data for LemonAid (751×coverage) and Tonic (4142×coverage) yielded double-stranded 167 749 bp and 168 103 bp linear genomes on single contigs for LemonAid and Tonic, with GC-content of 36.8 and 36.7 %, respectively. CheckV estimated the genomes were 100 % complete, with identified direct terminal repeats. Phage.ai classified LemonAid and Tonic as virulent with 70.5 and 71.5 % probability, respectively. There were no known AMR or virulence genes in any of the phages.

LemonAid has 252 genes and Tonic has 251 genes, 134 of which have predicted function in both (Fig S3a–b). There are major differences between the two phages in genes involved in host recognition and absorption, such as the long distal tail fibre gene (Fig. S4), which is 5307 bp long with 48 % average nucleotide identity between the two phages.

Analysis using ViPTree placed LemonAid and Tonic in a clade with other *A. baumannii* myoviruses, specifically in the family *Straboviridae* and the subfamily *Twarogvirinae*. Taxonomic analysis in VIRIDIC confirmed that LemonAid had <95 % intergenomic similarity with this related phage, and thus represents a new species in the genus *Lazarusvirus* in accordance with guidance from the International Committee of the Taxonomy of Viruses [65] (Fig. S5). Tonic has 95.5 % intergenomic similarity with LemonAid and is thus classified as a strain of the same species.

### Prophage induction during LemonAid infection

**Fig. 2.**
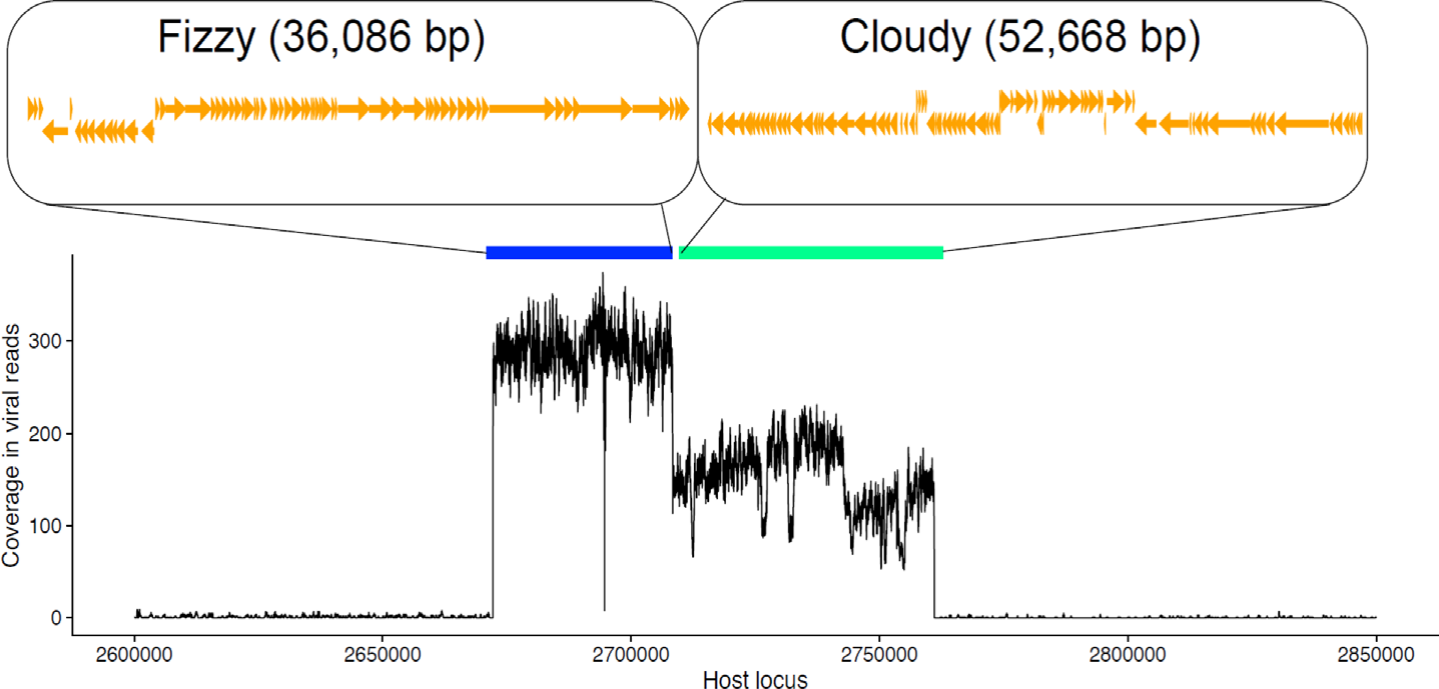
Alignment of LemonAid lysate reads against the host bacteria, *A. baumannii* NCTC 13420, showing high coverage of the two prophage Fizzy and Cloudy.

Sequencing of DNA from purified LemonAid lysate revealed the presence of two additional phage, named Cloudy and Fizzy. Phage.ai predicted that both Cloudy and Fizzy are temperate with 88 and 90 % probability, respectively. Annotations revealed a repressor protein in both and the phage genomes aligned exactly with the assembled *A. baumannii* genome (Fig. 2). Cloudy and Fizzy are both classified as siphoviruses by ViPTree and are widely distributed across diverse *A. baumannii* genomes (Fig. S6). Cloudy is 52 668 bp (13×coverage) and Fizzy is 38 086 bp (24×coverage). Alignment of raw LemonAid reads against the *A. baumannii* host genome revealed high coverage against both prophage regions (Fig. 2), consistent with prophage induction during a virulent infection.

### Phage resistance and virulence *in vitro*

**Fig. 3.**
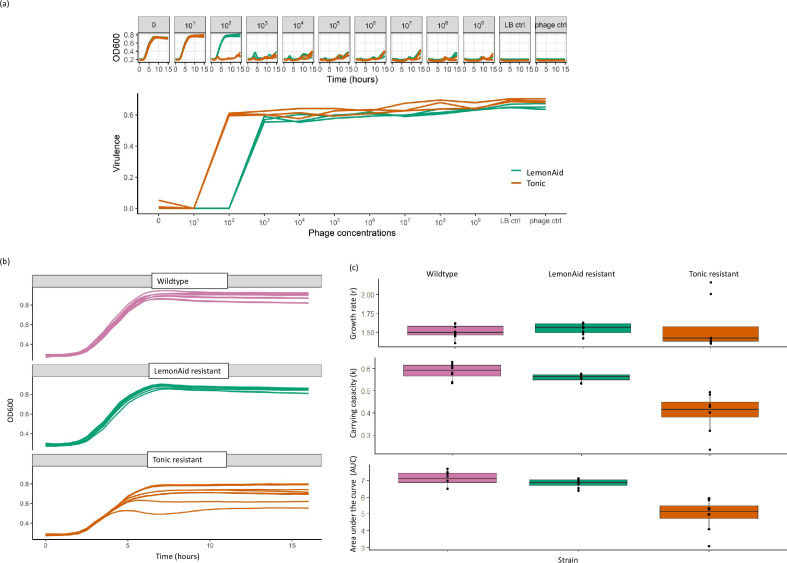
**(a**) Replicate (*N*=3) *in vitro* growth curves of *A. baumannii* NCTC 13240 WT infected with novel bacteriophage, LemonAid (4.6×10^9^) and Tonic (6.6×10^9^), in 1 : 10 serial dilution and the virulence of each phage against *A. baumannii*, calculated from the growth curves as 1- AUC (phage treatment)/AUC (no phage). (**b**) *In vitro* growth of *A. baumannii* NCTC 13420 and the phage-resistant strains (eight replicates). (c) Comparison of wild-type and phage-resistant *A. baumannii* variants: growth curve indices: AUC, *k* (carrying capacity) and *r* (growth rate). AUC=area under the curve. LB ctrl=LB broth with no bacteria or phage present. Phage ctrl=phage only at highest titre.

*In vitro*, LemonAid and Tonic suppressed *A. baumannii* growth at phage titres of 10^3^ or 10^2^, respectively, to 10^9^ p.f.u. ml^−1^, with a virulence index of 0.6, regardless of phage concentration. No measurable killing was observed at <10^2^ p.f.u. ml^−1^. Early regrowth of *A. baumannii* during the first 5 h was suppressed in a dose-dependent manner and eliminated at 10^9^ p.f.u. ml^−1^. At all titres, there was evidence of host regrowth by the end of the 15 h experiment (Fig. 3a). Indeed, after overnight culturing, cultures were turbid suggesting strong bacterial growth despite phage presence, and phage no longer formed plaques on their corresponding phage-resistant variant of *A. baumannii*, even at high titres of 10^8-9^ p.f.u. ml^−1^. Notably, there was cross-resistance, i.e. Tonic did not form plaques on LemonAid-resistant *A. baumannii*, and vice versa.

LemonAid and Tonic-resistant variants were cultured for further analysis. Compared to the wild-type *A. baumannii*, the phage-resistant variants showed no clear difference in colony morphology on solid agar. *In vitro*, grown without phage, the LemonAid-resistant variant was not different to the WT. The Tonic-resistant variant growth rate was more variable between replicates (Fig. 3b) and had reduced fitness (Fig. 3c) demonstrated by the significantly lower carrying capacity (*k*) and AUC compared to the wild-type (W=64, *p.adj* <0.001, effect size=0.84). However, there was no significant difference in growth rate (*r*) between the variants (Fig. 3c).

### Mechanisms of resistance

#### Genomic comparison of phage-resistant *A. baumannii* to the wild-type

**Fig. 4.**
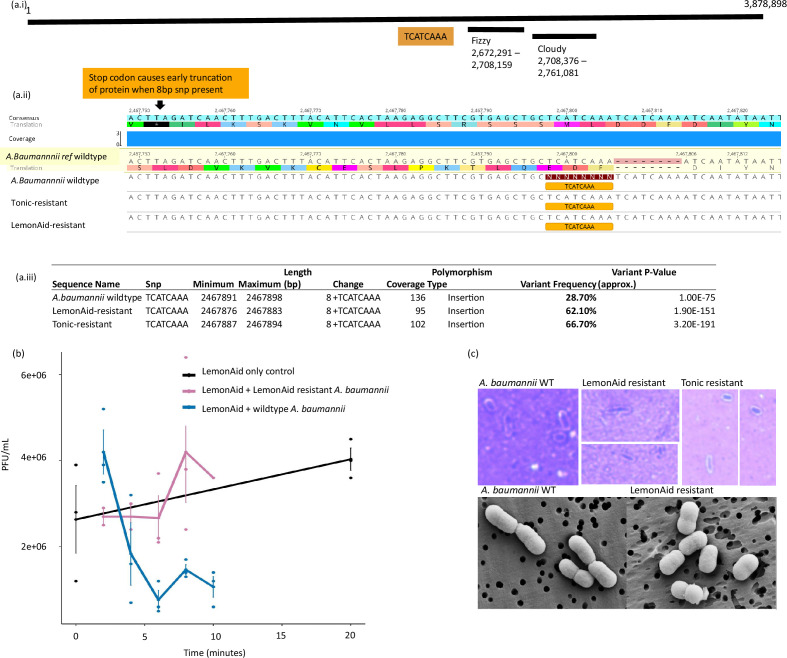
(a.i) Sketch representation of *A. baumannii* NCTC 13420 reference genome and the position of two active prophage (Fizzy and Cloudy) and an 8 bp insertion at 2 467 798 bp, (a.ii) an alignment of the wild-type and phage-resistant *A. baumannii* variants against the reference genome showing the sequence of the region of a gene of unknown function containing the 8 bp insertion and early truncation of the protein when the SNP is present and (a.iii) details of the insertion and its frequency in the different variants in Geneious. (b) Absorption assay: LemonAid p.f.u. ml^−1^ recorded during 20 min infections against *A. baumannii* WT (blue) and *A. baumannii* LemonAid-resistant variant (magenta), to estimate phage adsorption, (**c**) examples of cell staining and light microscopy, and SEM images of WT *A. baumannii* and phage-resistant variants.

The reference strain of *A. baumannii* NCTC 13420 was generated using long- and short-read sequencing and revealed a 3.88 Mb chromosome and two plasmids of 110 966 bp and 10 967 bp. *A. baumannii* NCTC 13420 is unlikely to carry a CRISPR system according to analysis using CRISPR-CAS ++software and Padloc (method S3). The former identified a single low-confidence spacer that did not match to the genomes of LemonAid or Tonic, while the latter did not identify a spacer region. Read coverage of the Minimap2 alignments of wild-type and phage-resistant variant short reads to the reference genome were: wild-type=× 205.7 (s.d.=40.1), LemonAid-resistant=× 141.8 (s.d.=30.1) and Tonic-resistant=× 140.8 (s.d.=30.9). A comparison of the LemonAid-resistant and Tonic-resistant variants to the reference wild-type *A. baumannii* revealed few genomic differences that were confirmed by all three tools employed for SNP calling, and no SNPs were called within known capsular polysaccharide (KL) or lipooligosaccharide outer core (OCL) genes (Table S2). However, the frequency of an 8 bp insertion mutation (TCATCAAA at 2 467 798 bp, producing a tandem repeat TCATCAAATCATCAAA) increased to 66.7 % in the LemonAid-resistant variant and 62.1 % in the Tonic-resistant variant, compared to 28.7 % in the wild-type (Fig. 4a, Table S2), and was confirmed by all three tools. When present, this 8 bp insertion introduces a stop codon 44 bp downstream of the insertion and likely causes a frame shift and potential early truncation of a protein of unknown function (Figs 4 and S7). The sequence TCATCAAA is commonly found throughout the *A. baumannii* genome (217 times), but the tandem repeat (i.e. TCATCAAATCATCAAA) was only found at the one location. Further informatic analysis of the protein carrying these mutations revealed no further information on its function (Table S3). Analysis of unmapped reads revealed no extra genes or plasmids in the resistant strains.

#### Adsorption assay and capsule structure

An adsorption assay revealed that LemonAid cannot adsorb to the LemonAid-resistant genotype (Fig. 4b), suggesting that the defence mechanism involves the phage-binding receptor protein or associated proteins. However, SEM and capsule staining techniques revealed no clear changes to the capsule surface (Fig. 4c).

### Resistance to phages comes with a cost to virulence *in vivo*

**Fig. 5.**
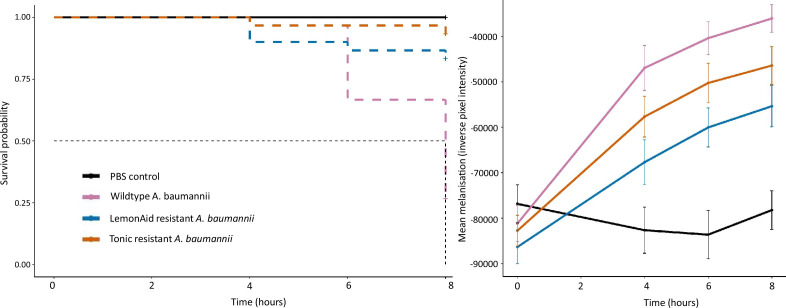
Survival (**a**) and melanization (**b**) of *G. mellonella* larvae inoculated with *A. baumannii* NCTC 13420 (magenta) and phage-resistant forms of the same strain (LemonAid-resistant – blue; Tonic-resistant – orange).

We tested if resistance to phage reduced the virulence of *A. baumannii* in a *G. mellonella* model and found the survival time of infected larvae increased significantly when inoculated with the phage-resistant variants, compared to the WT (Fig. 5a) [full model: χ^2^=55.6 on 3df, *P*<0.001; pairwise corrected *p*-value (BH) <0.001 for comparison of survival curves for LemonAid-resistant and Tonic-resistant variants versus WT *A. baumannii*]. End-point mortality of larvae at 8 h was 73.3 % (*N*=30) for wild-type infections, compared to 16 and 7 % (*N*=30) for LemonAid- and Tonic-resistant variants, respectively. In support of the survival data, melanization of larvae occurred significantly less in larvae inoculated with the phage-resistant strain (Fig. 5b, Table S4).

### LemonAid was not an effective therapeutic in *G. mellonella*

**Fig. 6.**
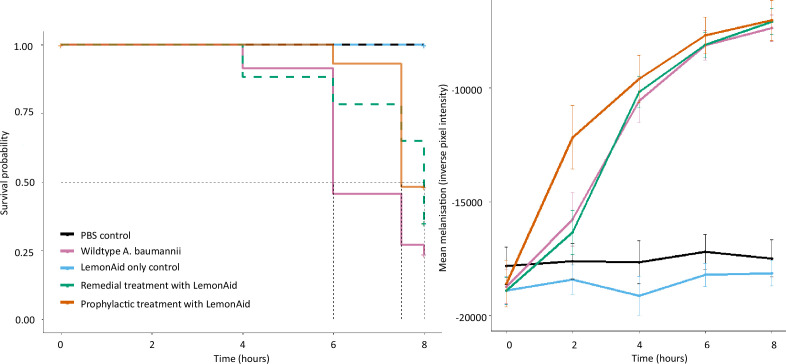
Comparison of (**a**) survival and (**b**) melanization across *G. mellonella* treatment groups: negative controls (1×PBS and phage-only), positive control (inoculation with *A. baumannii* only) at a dose of 4.6×10^6^ c.f.u., and remedial and prophylactic treatment with LemonAid (at a dose of 4.6×10^6^ p.f.u.) of *A. baumannii* inoculated larvae. The dotted lines in (a) represent the median survival time of each treatment group. Note, *A. baumannii* only and remedial treatment groups (*N*=60), prophylactic phage treatment (*N*=30).

Treating *A. baumannii* infected larvae*,* both remedially and prophylactically, with LemonAid significantly increased survival time [Fig. 6a; full model χ^2^=82.2 on 4df *p* = < 0.001; pairwise corrected *p*-value (BH) for remedial treatment=0.014 (*n*=60), and for prophylactic treatment=0.002 (*N*=30)]. 75 % (*N*=60) of untreated *A. baumannii* infected larvae died within 8 h, compared to 65 % (*N*=60) of the remedially treated and 50 % (*N*=30) of the prophylactically treated larvae. There was a significant difference in end-point mortality between prophylactically treated and untreated larvae but not remedially treated and untreated larvae (test of proportions: χ^2^=5.62 on 1df., *P*=0.03 and χ^2^=0.99 on 1df., *P*=0.32, respectively). As LemonAid enables the induction of prophages, Cloudy and Fizzy, during infection of *A. baumannii* NCTC-13420, the LemonAid inoculum likely contained low levels of these prophages, and likely induced further prophage excision during infection within the larvae. It is not clear what impact the presence or excision of prophages has on the treatment.

While LemonAid can increase survival of infected larvae when treated remedially and prophylactically, the larvae are not rescued from disease or death, thus the effect of phage treatment on the course of disease appears weak. Both phage-treated and untreated larvae melanized at the same rate (Fig. 6b, Table S5). There was no effect of larvae batch or experiment day on melanization data (Table S5). LemonAid did not affect survival or end-point mortality at a lower dose (LD50) of *A. baumannii* (Fig. S8). Note that we observed no deaths or malaise in negative control larvae inoculated with PBS or phage inoculum, or in no-stab controls.

Endotoxin testing revealed a filtered LemonAid lysate at a titre of 1×10^9^ p.f.u. ml^−1^ contained 3500 EU (endotoxin units) per ml. In preliminary work, larvae inoculated with 10 µl of LemonAid at 10^8^ p.f.u. ml^−1^ (equivalent to 3.5 EU per larva), demonstrated melanization. The titre of LemonAid used in the larval assays was 10^7^ p.f.u. ml^−1^, and a 10 µl dose would therefore have contained 0.35 EU. At this concentration of endotoxin, no melanization was observed in larvae receiving phage inoculum alone, indicating that at higher titres, endotoxin or other metabolites present in the phage filtrate were having a detrimental effect on the larvae.

## Discussion

The recent successes of compassionate phage treatment of patients with systemic AMR *A. baumannii* infections [21,23,66] have catalysed the search for potential phage candidates. Here we describe two strains of a novel, virulent myovirus species, isolated from environmental samples, that efficiently infects a carbapenem-resistant *A. baumannii* strain, reducing bacterial growth and, at high titres, suppressing the emergence of resistance *in vitro* during the 15 h growth experiment. However, after overnight culturing at lower titres resistance to phages consistently emerged, and we demonstrate in a *G. mellonella* larval model that resistance to LemonAid comes at a cost to *A. baumannii* virulence, in support of previous work [28]. While the mechanisms of resistance remain elusive but do not appear to be linked to reduction or loss of capsule, the phage-induced evolution of strains with lower virulence has potential benefits to health outcomes in patients. However, while survival of larvae was significantly increased, both remedial and prophylactic phage treatment failed to prevent morbidity or death in a *G. mellonella* model. The effective killing of *A. baumannii in vitro* with high titres of these novel phages versus the weak impact on larvae mortality *in vivo* highlights the need for *in vivo* testing of potential phage treatments.

LemonAid and Tonic are likely virulent phage (i.e. using the bacterial cell machinery to reproduce before lysing the cell) forming small but clear plaques on solid agar and killing bacterial cells in liquid broth. The genome contains no lysogenic genes, such as repressor genes that control the switch between lytic and lysogenic cycles and spannins and integrases that enable the phage to incorporate its genome into the host genome [67]. *Acinetobacter* harbour a high number of prophage (lysogenic phages incorporated into the host genome) [68]. We found two prophages in *A. baumannii* reference strain NCTC-13420, named Cloudy and Fizzy, are induced during infection with LemonAid. They contained no known AMR genes or virulence factors. While temperate phages are often avoided as candidates for phage therapy due to a potential risk of transduction of AMR genes and increased host fitness, it is likely that induction of prophage within a pathogenic bacterial target during treatment with virulent phages enhances bacterial killing. In addition, any phage products produced by propagating phages on the target host strain likely include induced prophages as well as virulent phages, highlighting the need for sequencing of phage products prior to use to assess their composition.

A number of studies have linked phage-resistance in *A. baumannii* to mutations in capsule genes [30,32] or reduced capsule production and avirulence [18,23]. However, in this study, resistance to LemonAid or Tonic was not correlated with visible alteration of the bacteria cells or capsule, and no mutations were observed within the capsular polysaccharide (KL) or lipooligosaccharide outer core (OCL) genes [69], suggesting a different, unknown mechanism associated with resistance and reduction in virulence.

*A. baumannii* resistance to both LemonAid and Tonic correlates with increased frequency, compared to the wild-type population, of an 8 bp insertion within a gene of unknown function that likely truncates the protein. Apart from this insertion, there were few genomic differences and none that were found independently in response to infection by both LemonAid and Tonic and confirmed by three methods of variant-calling. Thus, it is plausible that the increased frequency of this insertion provides resistance to these phages. The increased frequency of the insertion in the population could be caused by phase variation or by natural selection and fixation in the population. Phase variation causes resistance to phage infection by stochastic and reversible phase variation on/off switching of phage receptors, mediated by simple sequence repeats [25,70, 71]. Unfortunately, there is no direct information on the function of the gene or protein to date, the protein contains no transmembrane domains or signal peptides. As LemonAid cannot adsorb to the LemonAid-resistant variant, it is possible that the protein contains the phage-receptor site or is associated with the phage-receptor site. Further, the gene is found within a conserved group of genes that includes mupP 1, which is involved in the polysaccharide recycling pathway, and could play a role in altering wall components and affecting phage absorbance.

Despite the weak therapeutic effect of LemonAid and Tonic on larvae morbidity, these novel phages may provide value in phage therapy via their ability to reduce virulence of MDR *A. baummanii*. Phage therapy can work via direct killing of bacterial cells, but also via the attenuation of virulence, as the evolution of resistance against phage can cause fitness costs to the host bacteria [72,73]. In addition, evolved changes to the bacterial genome in response to phage can make the bacteria vulnerable to other phages and/or antibiotics (e.g. [30], thus successful treatment involving combinations of multiple phages used in succession, or a synergy with antibiotics, could improve therapy outcomes [74,75]. Thus, while LemonAid and Tonic did not prevent death in larvae directly, they reduced the virulence of *A. baumannii* and further research is required to discover their potential for treatment within antibiotic synergies and phage cocktails. Given the genetic similarity of LemonAid and Tonic, and the cross resistance they induce, we do not suggest they are used in cocktail together.

Compared to previous work using *A. baumannii* in *G. mellonella* models, *A. baumannii* NCTC 13420 appeared to be atypically virulent, with ~80 % mortality occurring in 8 h (compared to 16–48 h found in studies using similar doses [16,37]. This is likely due to variability in pathogenicity of strains but also the source and treatment of the larvae: diet and time of starvation affect survival of larvae because the fat body, which is reduced by storage of larvae without access to food, plays a role in immunity [76], internal comms.). For example, in our lab, larvae from a different source and used directly from food, i.e. no starvation, survived twice as long as the larvae in the current study when injected with the same strain and dose of *A. baumannii* (unpublished data). While other studies have found that phage therapy has rescued a proportion of larvae from infection [16,35, 37, 77, 78], in the current study, although life was prolonged, phage was unable to prevent death in a *G. mellonella* model. We hypothesize that endotoxins released by phage-mediated cell lysis may contribute to killing the larvae so that no amount of killing of the bacterial cells can prevent death. Indeed, high levels of endotoxins were found in the phage lysate concentrations used in the larvae assay.

The dose of *A. baumannii* injected into the larvae was high (10^6^ cells), however, at a reduced dose (10^5^ cells) LemonAid treatment did not impact end-point mortality, suggesting that there may be a density-dependency killing effect, i.e. the phage requires high numbers of cells to have an impact [79]. Given the evolution of resistance observed in *A. baumannii* in response to LemonAid and Tonic infection *in vitro*, it is not clear if the phages increase larvae survival by direct killing of bacterial cells or by selection of the bacteria to a less virulent, but phage-resistant form. It is possible that same resistance mechanism, and thus avirulence, would evolve *in vivo*, despite the additional pressures of the host immune system and spatial heterogeneity [31,80].

In conclusion, we have isolated and purified two strains of a novel phage species from environmental samples, with potential to treat a critically important nosocomial carbapenem-resistant *A. baumannii*. We demonstrate that phage-resistance leads to a loss of fitness and virulence of *A. baumannii* NCTC 13420. The mechanism of resistance and loss of virulence remains elusive but does not appear to be linked to capsule. We also show that LemonAid kills *A. baumannii* NCTC 13420 *in vitro* and *in vivo*, yet the effects *in vivo* are weak. Further research into the use of LemonAid and Tonic in phage cocktails and antibiotic synergies is necessary to find the best way to exploit these phages in therapy.

## supplementary material

10.1099/jmm.0.001829 Uncited Supplementary Material 1.

## References

[1] Murray CJ, Ikuta KS, Sharara F, Swetschinski L, Robles Aguilar G (2022). Global burden of bacterial antimicrobial resistance in 2019: a systematic analysis. The Lancet.

[2] Tacconelli E, Carrara E, Savoldi A, Harbarth S, Mendelson M (2018). Discovery, research, and development of new antibiotics: the WHO priority list of antibiotic-resistant bacteria and tuberculosis. Lancet Infect Dis.

[3] Public Health England N. (2022). Detection of bacteria with carbapenem-hydrolysing betat-lactamases (carbapenemases).

[4] Kyriakidis I, Vasileiou E, Pana ZD, Tragiannidis A (2021). *Acinetobacter baumannii* antibiotic resistance mechanisms. Pathogens.

[5] Jawad A, Seifert H, Snelling AM, Heritage J, Hawkey PM (1998). Survival of *Acinetobacter baumannii* on dry surfaces: comparison of outbreak and sporadic isolates. J Clin Microbiol.

[6] Mohd Sazlly Lim S, Zainal Abidin A, Liew SM, Roberts JA, Sime FB (2019). The global prevalence of multidrug-resistance among *Acinetobacter baumannii* causing hospital-acquired and ventilator-associated pneumonia and its associated mortality: a systematic review and meta-analysis. J Infect.

[7] Peleg AY, Seifert H, Paterson DL (2008). *Acinetobacter baumannii*: emergence of a successful pathogen. Clin Microbiol Rev.

[8] Czaplewski L, Bax R, Clokie M, Dawson M, Fairhead H (2016). Alternatives to antibiotics-a pipeline portfolio review. Lancet Infect Dis.

[9] McCallin S, Sacher JC, Zheng J, Chan BK (2019). Current state of compassionate phage therapy. Viruses.

[10] Uyttebroek S, Chen B, Onsea J, Ruythooren F, Debaveye Y (2022). The Lancet Infectious Diseases.

[11] Khatami A, Foley DA, Warner MS, Barnes EH, Peleg AY (2022). Standardised treatment and monitoring protocol to assess safety and tolerability of bacteriophage therapy for adult and paediatric patients (STAMP study): protocol for an open-label, single-arm trial. BMJ Open.

[12] Dedrick RM, Guerrero-Bustamante CA, Garlena RA, Russell DA, Ford K (2019). Engineered bacteriophages for treatment of a patient with a disseminated drug-resistant *Mycobacterium abscessus*. Nat Med.

[13] Dedrick RM, Smith BE, Cristinziano M, Freeman KG, Jacobs-Sera D (2023). Phage therapy of Mycobacterium infections: compassionate use of phages in 20 patients with drug-resistant mycobacterial disease. Clin Infect Dis.

[14] Young MJ, Hall LML, Merabishvilli M, Pirnay J-P, Clark JR (2023). Phage therapy for diabetic foot infection: a case series. Clin Ther.

[15] Oliveira H, Domingues R, Evans B, Sutton JM, Adriaenssens EM (2022). Genomic diversity of bacteriophages infecting the genus *Acinetobacter*. Viruses.

[16] Jeon J, Park JH, Yong D (2019). Efficacy of bacteriophage treatment against carbapenem-resistant *Acinetobacter baumannii* in Galleria mellonella larvae and a mouse model of acute pneumonia. BMC Microbiol.

[17] Leshkasheli L, Kutateladze M, Balarjishvili N, Bolkvadze D, Save J (2019). Efficacy of newly isolated and highly potent bacteriophages in a mouse model of extensively drug-resistant *Acinetobacter baumannii* bacteraemia. J Glob Antimicrob Resist.

[18] Regeimbal JM, Jacobs AC, Corey BW, Henry MS, Thompson MG (2016). Personalized therapeutic cocktail of wild environmental phages rescues mice from *Acinetobacter baumannii* wound infections. Antimicrob Agents Chemother.

[19] Grygorcewicz B, Roszak M, Golec P, Śleboda-Taront D, Łubowska N (2020). Antibiotics act with vb_abap_agc01 phage against *acinetobacter baumannii* in human heat‐inactivated plasma blood and galleria mellonella models.. Int J Mol Sci.

[20] LaVergne S, Hamilton T, Biswas B, Kumaraswamy M, Schooley RT (2018). Phage therapy for a multidrug-resistant *Acinetobacter baumannii* craniectomy site infection. Open Forum Infect Dis.

[21] Nir-Paz R, Gelman D, Khouri A, Sisson BM, Fackler J (2019). Successful treatment of antibiotic-resistant, poly-microbial bone infection with bacteriophages and antibiotics combination. Clin Infect Dis.

[22] Rao S, Betancourt-Garcia M, Kare-Opaneye YO, Swierczewski BE, Bennett JW (2022). Critically Ill patient with multidrug-resistant *Acinetobacter baumannii* respiratory infection successfully treated with intravenous and Nebulized bacteriophage therapy. Frontiers in Microbiology.

[23] Schooley RT, Biswas B, Gill JJ, Hernandez-Morales A, Lancaster J (2017). Development and use of personalized bacteriophage-based therapeutic cocktails to treat a patient with a disseminated resistant *Acinetobacter baumannii* infection. Antimicrob Agents Chemother.

[24] Tan X, Chen H, Zhang M, Zhao Y, Jiang Y (2021). Clinical experience of personalized phage therapy against carbapenem-resistant *Acinetobacter baumannii* lung infection in a patient with chronic obstructive pulmonary disease. Front Cell Infect Microbiol.

[25] Egido JE, Costa AR, Aparicio-Maldonado C, Haas PJ, Brouns SJJ (2022). Mechanisms and clinical importance of bacteriophage resistance. FEMS Microbiol Rev.

[26] Labrie SJ, Samson JE, Moineau S (2010). Bacteriophage resistance mechanisms. Nat Rev Microbiol.

[27] Alseth EO, Pursey E, Luján AM, McLeod I, Rollie C (2019). Bacterial biodiversity drives the evolution of CRISPR-based phage resistance Europe PMC funders group.. Nature.

[28] Mangalea MR, Duerkop BA (2020). Fitness trade-offs resulting from bacteriophage resistance potentiate synergistic antibacterial strategies. Infect Immun.

[29] Morris F, Dexter C, Kostoulias X, Uddin MI, Peleg A (2019). The mechanisms of disease caused by *Acinetobacter baumannii*. Front Microbiol.

[30] Gordillo Altamirano F, Forsyth JH, Patwa R, Kostoulias X, Trim M (2021). Bacteriophage-resistant *Acinetobacter baumannii* are resensitized to antimicrobials. Nat Microbiol.

[31] Liu M, Hernandez-Morales A, Clark J, Le T, Biswas B (2022). Comparative genomics of *Acinetobacter baumannii* and therapeutic bacteriophages from a patient undergoing phage therapy. Nat Commun.

[32] Wang X, Loh B, Gordillo Altamirano F, Yu Y, Hua X (2021). Colistin-phage combinations decrease antibiotic resistance in *Acinetobacter baumannii* via changes in envelope architecture. Emerg Microbes Infect.

[33] Tao Y, Duma L, Rossez Y (2021). Galleria mellonella as a good model to study *Acinetobacter baumannii* pathogenesis. Pathogens.

[34] Buchon N, Silverman N, Cherry S (2014). Immunity in Drosophila melanogaster--from microbial recognition to whole-organism physiology. Nat Rev Immunol.

[35] Wintachai P, Naknaen A, Pomwised R, Voravuthikunchai SP, Smith DR (2019). Isolation and characterization of Siphoviridae phage infecting extensively drug-resistant *Acinetobacter baumannii* and evaluation of therapeutic efficacy in vitro and in vivo. J Med Microbiol.

[36] Wintachai P, Phaonakrop N, Roytrakul S, Naknaen A, Pomwised R (2022). Enhanced antibacterial effect of a novel Friunavirus phage vWU2001 in combination with colistin against carbapenem-resistant *Acinetobacter baumannii*. Sci Rep.

[37] Zhou W, Feng Y, Zong Z (2018). Two new lytic bacteriophages of the *Myoviridae* family against carbapenem-resistant *Acinetobacter baumannii*. Front Microbiol.

[38] Turton JF, Kaufmann ME, Warner M, Coelho J, Dijkshoorn L (2004). A prevalent, multiresistant clone of *Acinetobacter baumannii* in Southeast England. J Hosp Infect.

[39] Olsen NS, Hendriksen NB, Hansen LH, Kot W (2020). A new high-throughput screening method for phages: enabling crude isolation and fast identification of diverse phages with therapeutic potential. PHAGE.

[40] Ewels P, Magnusson M, Lundin S, Käller M (2016). MultiQC: summarize analysis results for multiple tools and samples in a single report. Bioinformatics.

[41] Wick RR, Judd LM, Gorrie CL, Holt KE (2017). Unicycler: resolving bacterial genome assemblies from short and long sequencing reads. PLoS Comput Biol.

[42] Nayfach S, Camargo AP, Schulz F, Eloe-Fadrosh E, Roux S (2021). CheckV assesses the quality and completeness of metagenome-assembled viral genomes. Nat Biotechnol.

[43] Shaffer M, Borton MA, McGivern BB, Zayed AA, La Rosa SL (2020). DRAM for distilling microbial metabolism to automate the curation of microbiome function. Nucleic Acids Res.

[44] Bouras G, Nepal R, Houtak G, Psaltis AJ, Wormald PJ (2023). Pharokka: a fast scalable bacteriophage annotation tool. Bioinformatics.

[45] Seemann T (2014). Prokka: rapid prokaryotic genome annotation. Bioinformatics.

[46] Tynecki P, Guziński A, Kazimierczak J, Jadczuk M, Dastych J (2020). PhageAI - bacteriophage life cycle recognition with machine learning and natural language processing. Bioinformatics.

[47] Darling ACE, Mau B, Blattner FR, Perna NT (2004). Mauve: multiple alignment of conserved genomic sequence with rearrangements. Genome Res.

[48] Chen L, Yang J, Yu J, Yao Z, Sun L (2005). VFDB: a reference database for bacterial virulence factors. Nucleic Acids Res.

[49] Alcock BP, Raphenya AR, Lau TTY, Tsang KK, Bouchard M (2020). CARD 2020: antibiotic resistome surveillance with the comprehensive antibiotic resistance database. Nucleic Acids Res.

[50] Yukgehnaish K, Rajandas H, Parimannan S, Manickam R, Marimuthu K (2022). PhageLeads: rapid assessment of phage therapeutic suitability using an ensemble machine learning approach. Viruses.

[51] Nishimura Y, Yoshida T, Kuronishi M, Uehara H, Ogata H (2017). ViPTree: the viral proteomic tree server. Bioinformatics.

[52] Moraru C, Varsani A, Kropinski AM (2020). VIRIDIC-a novel tool to calculate the intergenomic similarities of prokaryote-infecting viruses. Viruses.

[53] Wickham H (2016). Ggplot2: elegant Graphics for data analysis (use R!).

[54] Storms ZJ, Teel MR, Mercurio K, Sauvageau D (2020). The virulence index: a metric for quantitative analysis of phage virulence. PHAGE.

[55] Sprouffske K, Wagner A (2016). Growthcurver: an R package for obtaining interpretable metrics from microbial growth curves. BMC Bioinformatics.

[56] Kassambara A (2023). Rstatix: pipe-friendly framework for basic statistical tests, R package version 0.7.2.

[57] Hothorn T, Hornik K, Wiel M van de, Zeileis A (2008). Journal of Statistical Software Implementing a Class of Permutation Tests: The coin Package. http://www.jstatsoft.org/.

[58] Li H (2018). Minimap2: pairwise alignment for nucleotide sequences. Bioinformatics.

[59] Danecek P, Bonfield JK, Liddle J, Marshall J, Ohan V (2021). Twelve years of SAMtools and BCFtools. Gigascience.

[60] Deatherage DE, Barrick JE (2014). Identification of mutations in laboratory-evolved microbes from next-generation sequencing data using breseq. Methods Mol Biol.

[61] Hughes RB, Smith AC (2007). American Society for Microbiology.

[62] Champion OL, Cooper IAM, James SL, Ford D, Karlyshev A (2009). Galleria mellonella as an alternative infection model for *Yersinia pseudotuberculosis*. Microbiology.

[63] Therneau T (2023). A Package for Survival Analysis in R. R package version 3.5-5.

[64] Bates D, Machler M, Bolker B, Walker S (2015). Fitting linear mixed-effects models using Lme4. J Stat Softw.

[65] Adriaenssens EM, Brister JR (2017). How to name and classify your phage: an informal guide. Viruses.

[66] Law N, Logan C, Yung G, Furr CLL, Lehman SM (2019). Successful adjunctive use of bacteriophage therapy for treatment of multidrug-resistant *Pseudomonas aeruginosa* infection in a cystic fibrosis patient. Infection.

[67] Canchaya C, Proux C, Fournous G, Bruttin A, Brüssow H (2003). Prophage genomics. Microbiol Mol Biol Rev.

[68] Loh B, Chen J, Manohar P, Yu Y, Hua X (2020). A biological inventory of prophages in *A. baumannii* genomes reveal distinct distributions in classes, length, and genomic positions.. Front Microbiol.

[69] Wyres KL, Cahill SM, Holt KE, Hall RM, Kenyon JJ (2020). Identification of *Acinetobacter baumannii* loci for capsular polysaccharide (KL) and lipooligosaccharide outer core (OCL) synthesis in genome assemblies using curated reference databases compatible with *Kaptive*. Microb Genom.

[70] Sandhu SK, Bayliss CD, Morozov AY (2021). How does feedback from phage infections influence the evolution of phase variation in Campylobacter?. PLoS Comput Biol.

[71] Tipton KA, Dimitrova D, Rather PN (2015). Phase-variable control of multiple phenotypes in *Acinetobacter baumannii* strain AB5075. J Bacteriol.

[72] Chan BK, Stanley GL, Kortright KE, Modak M, Ott IM (2023). Personalized inhaled bacteriophage therapy decreases multidrug-resistant *Pseudomonas aeruginosa* header phage therapy reduces Pseudomonas bacterial load and virulence. MedRxiv.

[73] Gurney J, Pradier L, Griffin JS, Gougat-Barbera C, Chan BK (2020). Phage steering of antibiotic-resistance evolution in the bacterial pathogen, *Pseudomonas aeruginosa*. Evol Med Public Health.

[74] Abedon ST, Danis-Wlodarczyk KM, Wozniak DJ (2021). Phage cocktail development for bacteriophage therapy: toward improving spectrum of activity breadth and depth. Pharmaceuticals.

[75] Łusiak-Szelachowska M, Międzybrodzki R, Drulis-Kawa Z, Cater K, Knežević P (2022). Bacteriophages and antibiotic interactions in clinical practice: what we have learned so far. J Biomed Sci.

[76] Wojda I (2017). Immunity of the greater wax moth *Galleria mellonella*. Insect Sci.

[77] Beeton ML, Alves DR, Enright MC, Jenkins ATA (2015). Assessing phage therapy against *Pseudomonas aeruginosa* using a *Galleria mellonella* infection model.. Int J Antimicrob Agents.

[78] Nale JY, Vinner GK, Lopez VC, Thanki AM, Phothaworn P (2021). An optimized bacteriophage cocktail can effectively control *Salmonella in vitro* and in Galleria mellonella. Front Microbiol.

[79] Payne R (2000). Clinical Pharmacology & Therapeutics.

[80] Castledine M, Padfield D, Sierocinski P, Pascual JS, Hughes A (2022). Parallel evolution of *Pseudomonas aeruginosa* phage resistance and virulence loss in response to phage treatment in vivo and in vitro. Elife.

