# Supplementary material for: Resistance to bacteriophage incurs a cost to virulence in drug-resistant Acinetobacter baumannii
Source: J Med Microbiol. 2024 May 14;73(5):001829. doi: 10.1099/jmm.0.001829 (PMC11170128; doi:10.1099/jmm.0.001829)
Supplement: Uncited Supplementary Material 1. [file jmm-73-01829-s001.pdf]

Supplementary material for ‘Resistance to bacteriophage incurs a cost to virulence in drug resistant *Acinetobacter baumannii*‘

Supplementary figures

Figure S1. Galleria larvae with arrows showing the left and right proleg injection sites.

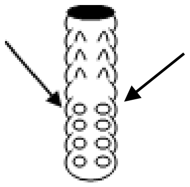

Figure S2. a) Green plates (Biosystems technologies) used in Galleria experiments for melanisation quantification, and the light distribution across the plate during imaging. b) Background signal from 50 PBS larvae in a 50 well plate showing the variation across the plate, and an example layout of treatment groups within plates to account for changes in light across plate and how distribution of treatment groups randomises these changes (colours represent each row) (b,c)

a)

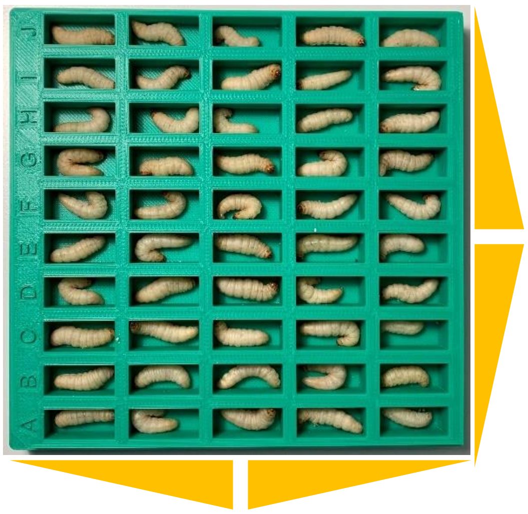

b)

	1	2	3	4	5
J	PBS + PBS	PBS + PBS	PBS + PBS	PBS + PBS	PBS + PBS
I	Bacteria + PBS	Bacteria +PBS	Bacteria	Bacteria	Bacteria
H	PBS + phage	PBS + phage	PBS + phage	PBS + phage	PBS + phage
G	Bacteria + phage	Bacteria + phage	Bacteria + phage	Bacteria + phage	Bacteria + phage
F	Treatment 5?	Treatment 5?	Treatment 5?	Treatment 5?	Treatment 5?
E	PBS + PBS	PBS + PBS	PBS + PBS	PBS + PBS	PBS + PBS
D	Bacteria + PBS	Bacteria + PBS	Bacteria + PBS	Bacteria + PBS	Bacteria + PBS
C	PBS + phage	PBS + phage	PBS + phage	PBS + phage	PBS + phage
B	Bacteria + phage	Bacteria + phage	Bacteria + phage	Bacteria + phage	Bacteria + phage
A	Treatment 5?	Treatment 5?	Treatment 5?	Treatment 5?	Treatment 5?

c)

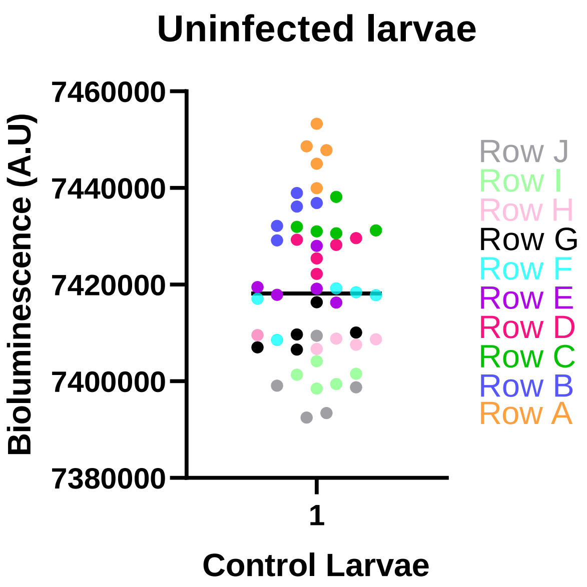

Figure S3. a) Annotated LemonAid and b) Tonic genome using Pharokka

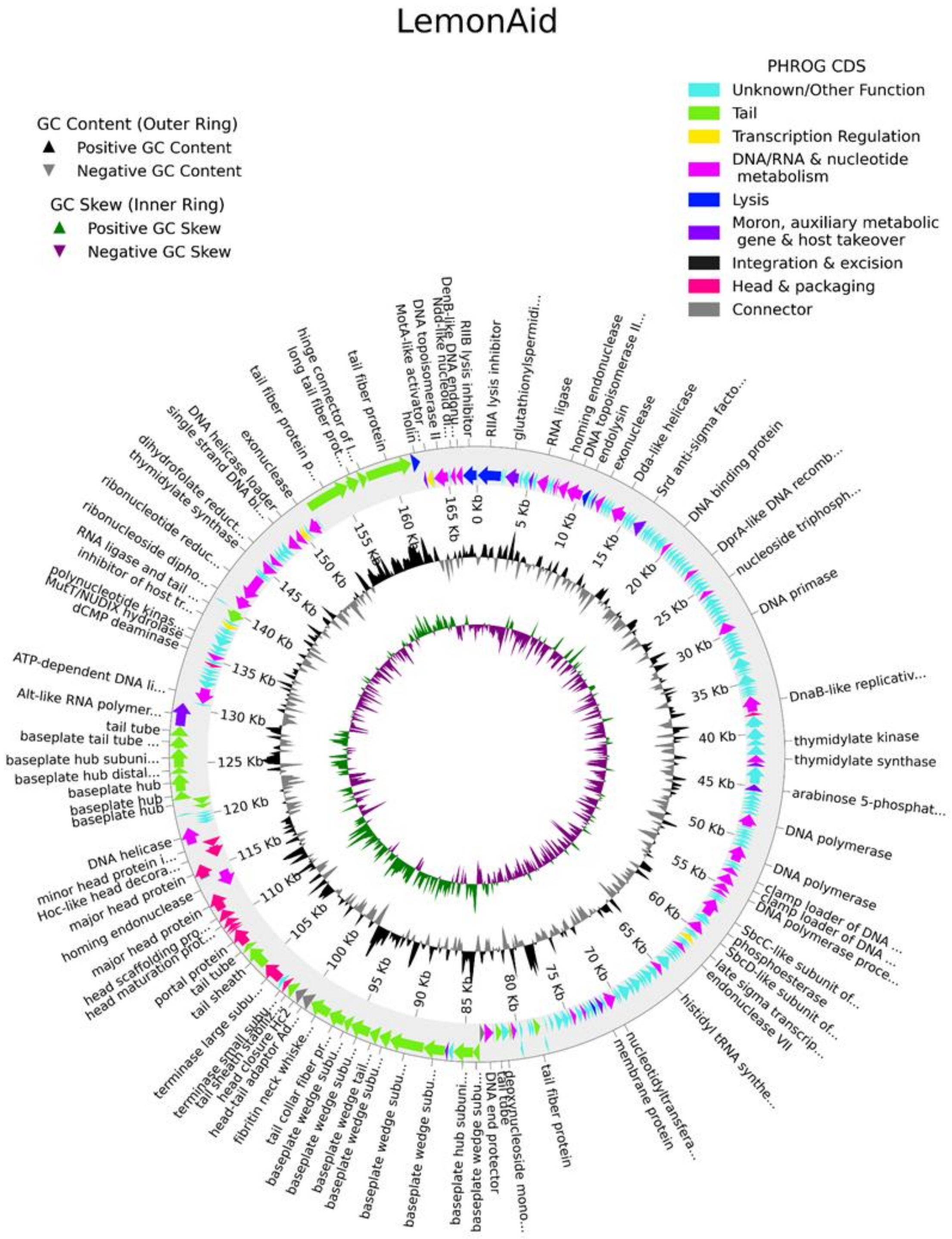

b)

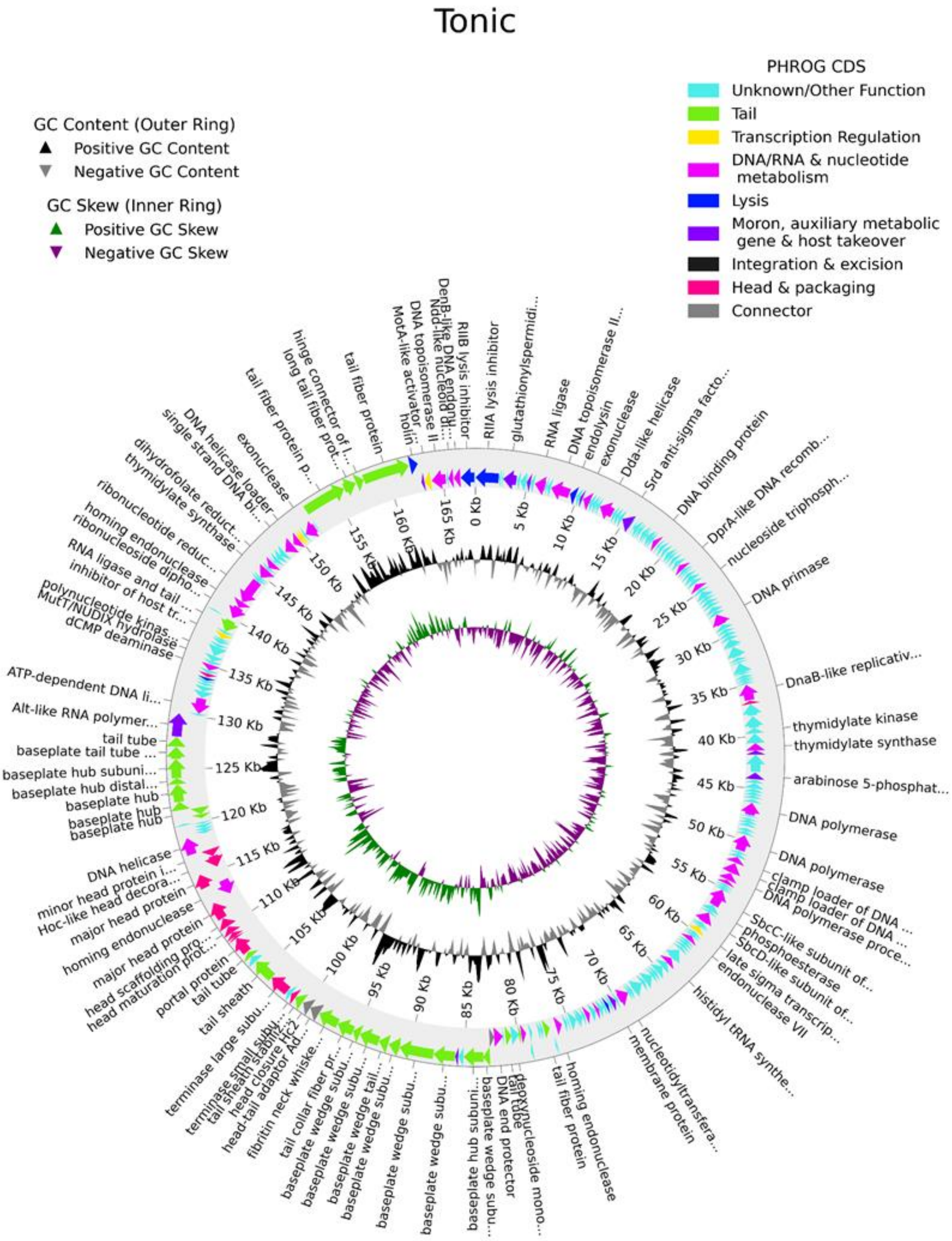
Tonic
GC Content (Outer Ring)
Positive GC Content
Negative GC Content
GC Skew (Inner Ring)
Positive GC Skew
Negative GC Skew
PHROG CDS
Unknown/Other Function
Tail
Transcription Regulation
DNA/RNA & nucleotide metabolism
Lysis
Moron, auxiliary metabolic gene & host takeover
Integration & excision
Head & packaging
Connector
0 Kb
5 Kb
10 Kb
15 Kb
20 Kb
25 Kb
30 Kb
35 Kb
40 Kb
45 Kb
50 Kb
55 Kb
60 Kb
65 Kb
70 Kb
75 Kb
80 Kb
85 Kb
90 Kb
95 Kb
100 Kb
105 Kb
110 Kb
115 Kb
120 Kb
125 Kb
130 Kb
135 Kb
140 Kb
145 Kb
150 Kb
155 Kb
160 Kb
165 Kb
RIIA lysis inhibitor
glutathionylspermidi...
RNA ligase
DNA topoisomerase II...
endolysin
exonuclease
Dda-like helicase
Srd anti-sigma facto...
DNA binding protein
DprA-like DNA recomb...
nucleoside triphosph...
DNA primase
DnaB-like replicativ...
thymidylate kinase
thymidylate synthase
arabinose 5-phosphat...
DNA polymerase
DNA polymerase
clamp loader of DNA ...
clamp loader of DNA ...
DNA polymerase proce...
SbcC-like subunit of...
phosphoesterase
SbcD-like subunit of...
late sigma transcrip...
endonuclease VII
histidyl tRNA synthe...
nucleotidyltransfera...
membrane protein
homing endonuclease
tail fiber protein
deoxynucleoside mono...
tail tube
DNA end protector
baseplate wedge subu...
baseplate hub subuni...
baseplate wedge subu...
baseplate wedge subu...
baseplate wedge subu...
baseplate wedge tail...
baseplate wedge subu...
baseplate wedge subu...
tail collar fiber pr...
fibritin neck whiske...
head-tail adaptor Ad...
head closure Hc2
tail sheath stabiliz...
terminase small subu...
terminase large subu...
tail sheath
tail tube
portal protein
head maturation prot...
head scaffolding pro...
major head protein
homing endonuclease
major head protein
Hoc-like head decora...
minor head protein i...
DNA helicase
baseplate hub
baseplate hub
baseplate hub
baseplate hub distal...
baseplate hub subuni...
baseplate tail tube ...
tail tube
Alt-like RNA polymer...
ATP-dependent DNA li...
dCMP deaminase
MutT/NUDIX hydrolase
polynucleotide kinas...
inhibitor of host tr...
RNA ligase and tail ...
ribonucleoside dipho...
homing endonuclease
ribonucleotide reduc...
thymidylate synthase
dihydrofolate reduct...
single strand DNA bi...
DNA helicase loader
exonuclease
tail fiber protein p...
long tail fiber prot...
hinge connector of l...
tail fiber protein
MotA-like activator...
holin
DNA topoisomerase II
DenB-like DNA endonu...
Ndd-like nucleoid di...
RIIB lysis inhibitor

Figure S4. Pairwise comparison of LemonAid and Tonic: 87% nucleotide pairwise identity across length, highlighting differences across genes (Geneious, Mauve, (Darling et al., 2004))

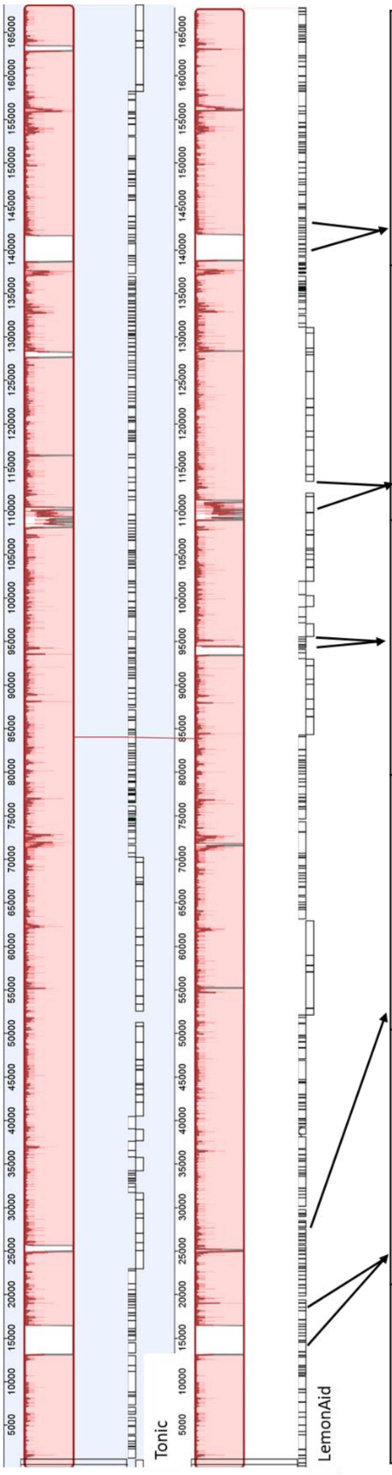

N genes affected	4	1	1	1	3
Name gene(s)	Baseplate wedge and hub subunits, tail lysozyme genes and a gene of unknown function	Additional putative homing nuclease in A2 but not in LemonAid	DNA topoisomerase large subunit	Long distal tail fibre	DNA ligase, RNA-polymerase, baseplate tail tube initiator
Length	16017bp region	594bp	5769bp	5322bp	7726bp region
Pairwise identity (%)	56.7	na	61.9	48.2	20.8

Figure S5. Viridic intergenomic similarity of LemonAid and Tonic against the 13 most similar phage genomes when searched against the LemonAid genome using NCBI Blast. The number indicates % intergenomic similarity, anything <95% is considered a separate species. Thus, LemonAid and Tonic are two variants of the same novel species.

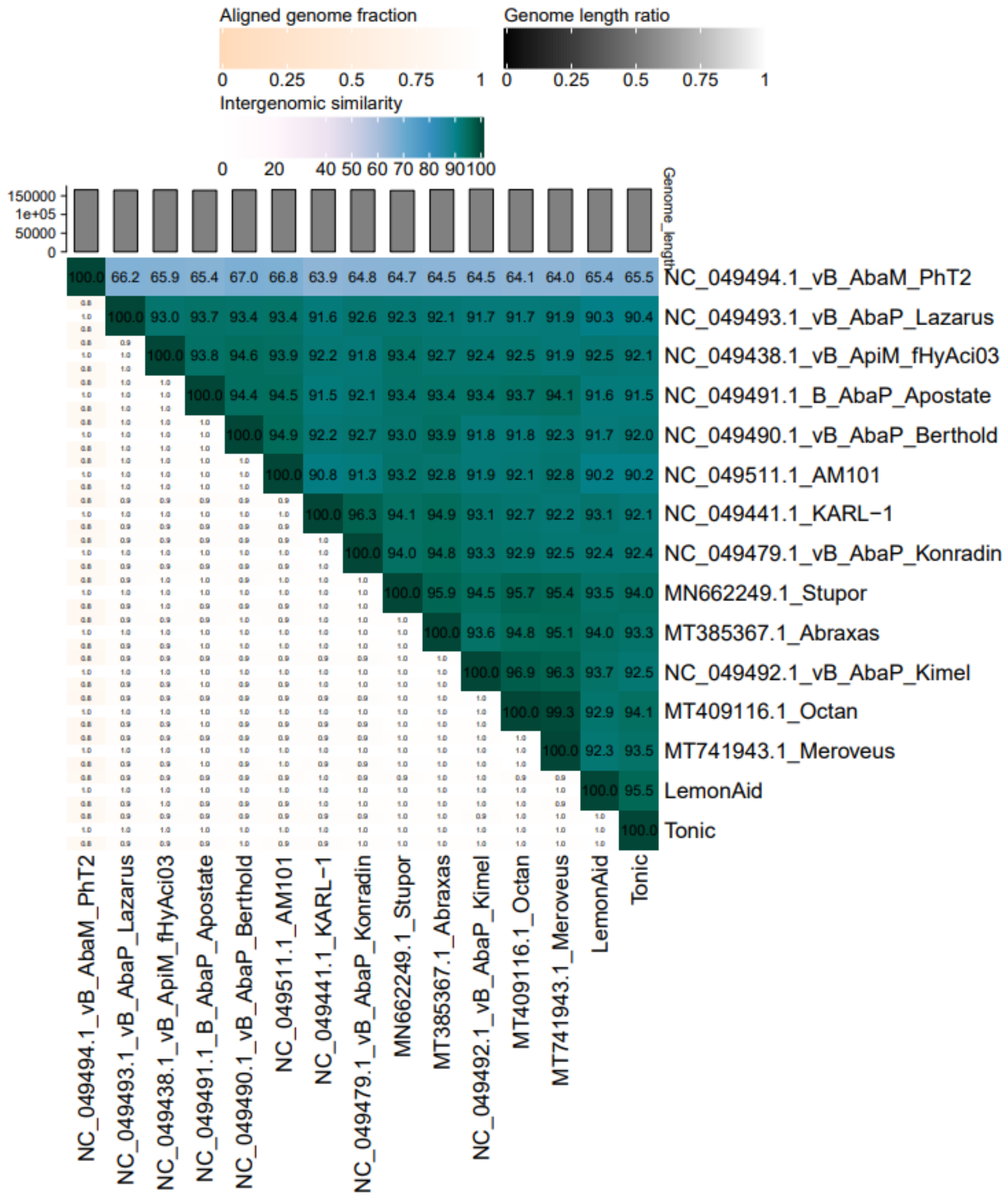

Figure S6. Distribution of two prophage, Fizzy and Cloudy, that were induced from host A. baumannii NCTC 13420 when infected with the virulent phage LemonAid. The tree is mid-point rooted. Fizzy is marked in red, Cloudy in green. It is worth noting that the phages appear to share the same attB site, integrating just downstream of the 6S RNA (ssrS) gene. Interestingly, Cloudy creates a 60 bp repeat at both ends of the prophage region that overlaps the ssrS sequence. It is possible this enables the second clade of prophages (e.g Fizzy) to integrate at the right repeat sequence, hence we observe tandem insertion of two prophages.

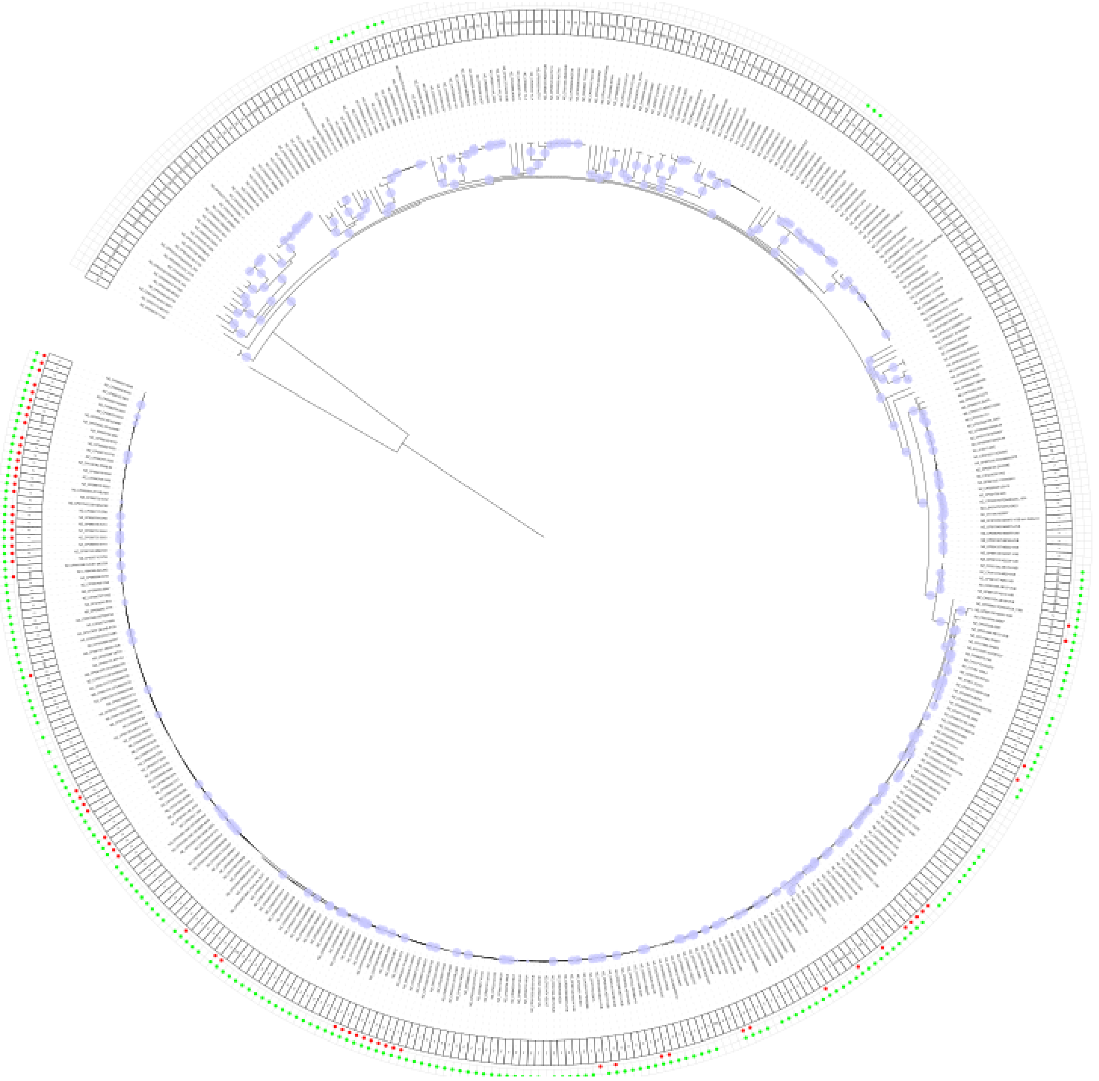

Figure S7. Protein of unknown function in full (green) and truncated (blue) when an 8 bp insertion is present

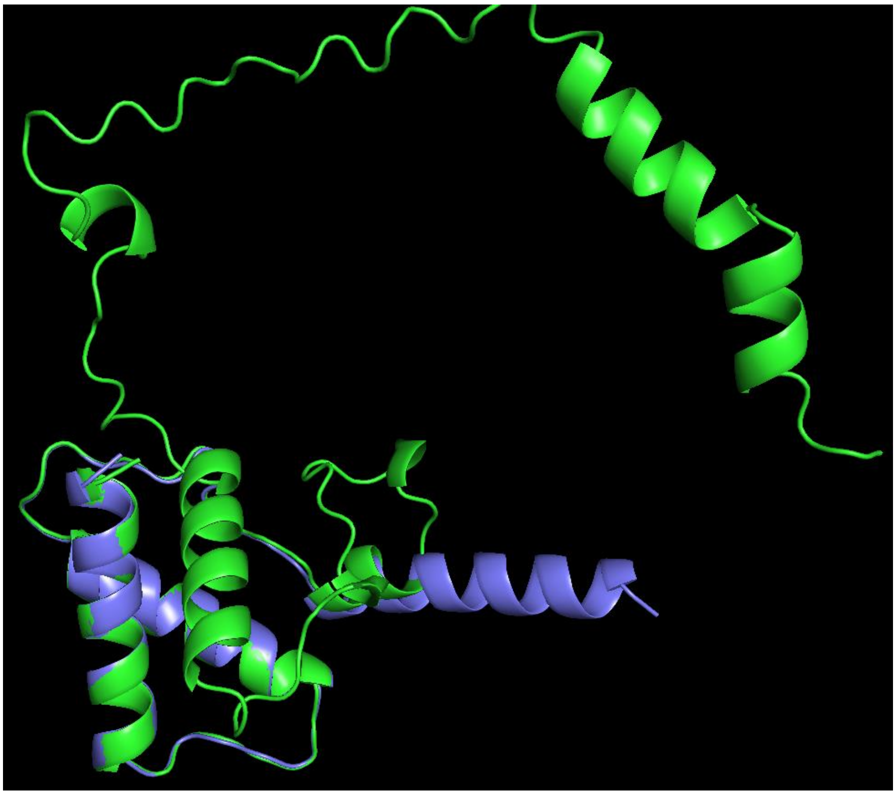

Figure S8. Galleria larvae infection experiment with reduced dose of *A. baumannii* (4 x10^5 cells). There was no difference between phage treatment (green) and *A. baumannii* positive control (magenta). Note: data collection was not taken between 12 and 24 hours. Kaplan Meier survival curve and model $\chi^2 = 89.6$, 3d.f, p = 0.001, corrected p-value for pairwise comparison between A. baumannii positive control and LemonAid treated larvae = 0.23, ns).

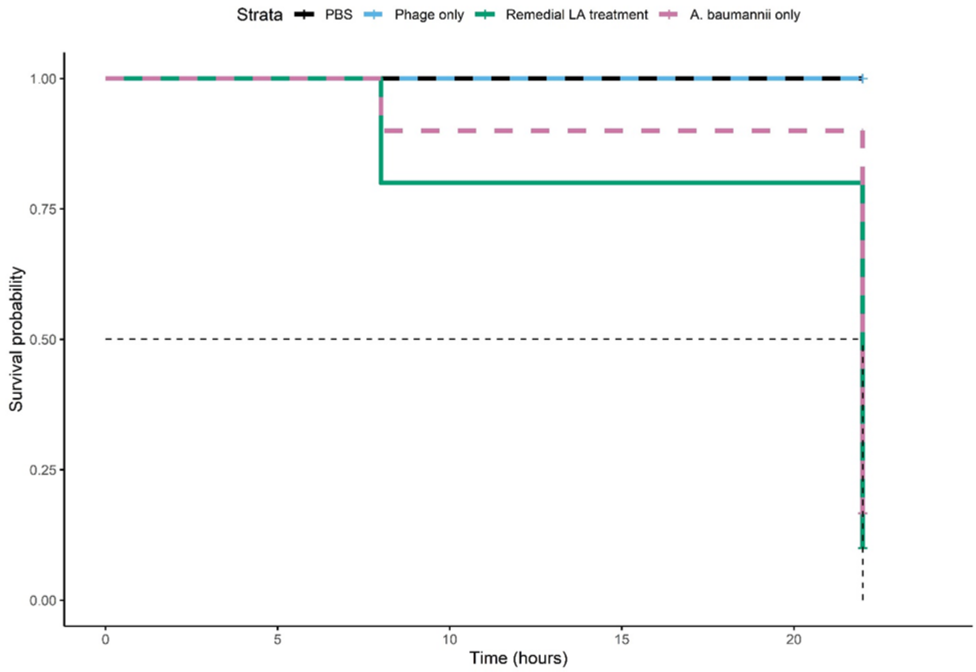

Supplementary tables

Table S1. Treatment groups and injection protocol for efficacy of LemonAid against A. baumannii.

Treatment (N)	1st injection	2nd Injection
A.baumannii positive control (60)	A. baumannii 10ul	1xPBS 10ul
Phage remedial treatment (60)	A. baumannii 10ul	LemonAid 10ul
Phage prophylactic treatment (30)	Lemonaid 10ul	A. baumannii 10ul
Phage negative control (60)	LemonAid 10ul	1xPBS 10ul
PBS negative control (60)	1xPBS 10ul	1xPBS 10ul

Table S2. Table of snps and insertions found by three methods of variant calling (Geneious, BCFtools and Breseq) when aligning the wildtype and phage resistant (LemonAid- or Tonic-resistant) variants short reads back to the reference genome. Only the 8bp insertion TCATCAAA was confirmed by all three methods.

Variant	Mutation	Position	Geneious	Freq in variant (%)	BCFtools	Freq in variant	Breseq	Freq	Gene product
Tonic	TCATCAAA	2,467,798	Yes	66.70	Yes	10,9:24,23	Yes, evidence of new junction	78	Hyp protein
LemonAid	TCATCAAA	2,467,798	Yes	62.10	Yes	11,12:28,31		75.2	
LemonAid	C to T	788,399	Yes	49.90	No	Na	No	Na	Hyp protein
LemonAid	C to A	2,358,425	Yes	30.80	No	Na	No	Na	Cytochrome b561
Tonic	AACAGAG	3,156,281	No	Na	Yes	6,4:13,18	Yes	89	Adenosine deaminase (coding)
LemonAid	AACAGAG	3,156,316 (breseq) 3,156,281 (vcf)	No	Na	Yes	3,7:13,12	Yes	63	Adenosine deaminase (intergenic)
LemonAid	TGAACC (11-22)	1,493,581	no	na	no	Na	yes	38.30	Holiday junction ATP-dependent DNA helicase RuvB (coding)

Table S3. Results of informatics search on hypothetical protein of interest

Informatics tool	Information
InterPro	No hits
HMMER	No hits
JackHammer	No hits
NCBI BLASTP	No known function in the top 1000 hits against NR (min 55% AAI)
ESMFold	V. low or Low pLDDT scores for all amino acids
Signal P 6.0	No signal peptides
STRING v 11.5	No known interactions
ScanProsite	No hit
CATH	No functional domains
CD-Search	No conserved domains
ColabFold v1.5.2 AlphaFold2	Structure found but the protein on which it is based has no known function

Table S4. Full linear model showing the effect of treatment, time and batch on melanisation of larvae in the phage-resistant assay, where the reference group is *A. baumannii* positive control

	Estimate	Standard error	t value	p value
Intercept	8434.4	247.5	34.1	0.00
LemonAid resistant strain	516.5	285.8	1.81	0.07
Tonic resistant strain	156.6	285.8	0.55	0.58
Time 4 hrs	-3421.7	285.8	-11.9	0.00
Time 6 hrs	-4076.13	285.83	-14.26	0.00
Time 8 hrs	-4509.6	285.83	-15.78	0.00
Batch	-157.94	71.46	-2.21	0.03
**Interaction treatment:time**				
Lemonaid-resistant:4hrs	1556.52	404.22	3.85	0.00
Tonic-resistant:4hrs	915.00	404.22	2.26	0.02
Lemonaid-resistant:6hrs	1447.03	404.22	3.58	0.00
Tonic-resistant:6hrs	829.37	404.22	2.05	0.04
Lemonaid-resistant:8hrs	1412.03	404.22	3.49	0.00
Tonic-resistant:8hrs	878.08	404.22	2.17	0.03

Table S5. Full linear model showing the effect of treatment, time, batch and experiment on melanisation of larvae in the phage treatment assay, with PBS negative control as reference group.

Variable	Estimate	Standard error	t value	p value
Intercept	17753.97	433.17	40.99	0.00
Treatment –*A. baumannii* positive	917.56	536.83	1.712	0.09
Treatment – LemonAid negative	1080.38	533.83	2.024	0.04
Treatment – Remedial treatment	1092.59	533.83	2.05	0.04
Treatment – Prophylactic treatment	826.12	659.26	1.25	0.21
Time 2hrs	-211.27	533.83	-0.40	0.69
Time 4hrs	-168.89	533.83	-0.31	0.75
Time 6hrs	-615.78	533.83	-1.15	0.24
Time 8hrs	-327.91	533.83	-0.61	0.54
Batch	58.04	97.46	0.60	0.55
Experiment	-96.54	169.17	-0.57	0.57
**Interactions treatment:time**				
Treatment A. baumannii:2hrs	-2748.55	758.14	-3.625	0.00
Treatment LemonAid:2hrs	-268.84	754.95	-0.356	0.72
Treatment remedial:2hrs	-2356.68	754.95	-3.12	0.00
Treatment prophylactic:2hrs	-6219.82	924.62	-6.73	0.00
Treatment A. baumannii:4hrs	-8003.49	758.14	-10.56	0.00
Treatment LemonAid:4hrs	401.54	754.95	0.532	0.59
Treatment remedial:4hrs	-8577.88	754.95	-11.36	0.00
Treatment prophylactic:4hrs	-8827.63	924.62	-9.55	0.00
Treatment A. baumannii:6hrs	-10007.06	758.14	-13.20	0.00
Treatment LemonAid:6hrs	-78.08	754.95	-0.10	0.92
Treatment remedial:6hrs	-10198.52	754.95	-13.51	0.00
Treatment prophylactic:6hrs	-10286.12	924.62	-11.13	0.00
Treatment A. baumannii:8hrs	-11034.24	758.14	-14.55	0.00
Treatment LemonAid:8hrs	-436.93	754.95	-0.58	0.56
Treatment remedial:8hrs	-11504.45	754.95	-15.24	0.00
Treatment prophylactic:8hrs	-11244.32	924.62	-12.16	0.00

Supplementary methods

Method S1 – Plaque assays and producing an axenic phage

Plaque assays: The soft-agar overlay technique (Kropinski et al., 2009) was used to determine if any *A. baumannii* phage were present in the enriched samples. Briefly, 30mL of 1% LB bottom agar, containing $CaCl_2$ and $MgCl_2$ at a final concentration of 10mM, was poured into a petri-dish, 3mL of 0.65% LB top agar was mixed with 1mL exponential phase (0.6 OD_{600}) *A. baumannii* (strain NCTC 13420) and 1mL of sample filtrate, and poured onto the solidified bottom agar plate and allowed to cool. Plates were incubated overnight at 37°C and then examined for the presence of plaques. Cores of the resulting plaques were taken and suspended in 100µL SM buffer (containing 100 mM NaCl, 50 mM Tris-HCl; 8 mM $MgSO_4{\cdot}H2O$). Core suspensions were serially diluted 1:10 and 10µl of each dilution was spotted onto 0.65% top agar LB seeded with 1mL 0.6 OD_{600} *A. baumannii*, using the overlay method. A core of a solitary plaque was taken, suspended in SM buffer and serially diluted. This process was repeated three times before selecting a plaque to take forward in experiments, to ensure the phages were axenic.

- Spot assays to determine resistance to phage

Bottom agar plates were poured and set as described above. 1mL of *A. baumannii* culture at 0.6 OD_{600} was added to 3mL of warm, liquid 0.65% LB top agar and poured onto the solidified bottom agar plate. After the top agar had been allowed to set, 10 µL of phage lysate were spotted onto the top agar and allowed to dry, before being incubated overnight at 37°C, and plaques counted the next day.

Method S2 - The distribution of prophage

To identify phage isolates in GenBank with sequence similarity, BLASTN searches were performed limited to the class *Caudoviricetes* (taxid: 2731619)

Cloudy

Accession	Name	Query coverage	Percentage identity
MZ675741.1	Acinetobacter phage Ab1656-2	72%	99.33%
KT588073.1	Acinetobacter phage Ab105-3phi	58%	97.43%
MZ514874.1	Acinetobacter phage Ab105-2phideltaCI404ad	50%	89.63%
KT588075.2	Acinetobacter phage Ab105-2phi	50%	99.69%

Fizzy

Accession	Name	Query coverage	Percentage identity
MT344105.1	Acinetobacter phage fLi-Aba03	39%	96.55%
MT344104.1	Acinetobacter phage fLi-Aba02	39%	96.55%
MT344103.1	Acinetobacter phage fEg-Aba01	39%	96.55%

To assess the distribution of prophage clades represented by Fizzy and Cloudy, BLASTN was performed against a dataset of 420 complete *Acinetobacter baumannii* genomes where putative prophage regions had been predicted using PhiSpy (https://github.com/linsalrob/PhiSpy, Turner, unpublished data). Presence of either phage was determined using a strict inclusion threshold of ≥95 sequence similarity. These data were used to annotate a core genome phylogeny created using FastTree with 100 bootstraps from a MAFFT (Katoh & Standley, 2013)core gene alignment produced by Roary (Page et al., 2015). Using a recombination naïve approach, Pasteur sequence type was predicted for each genome using mlst (https://github.com/tseemann/mlst) and the tree was annotated using ITOL (Letunic & Bork, 2021) .

Method S3 – Short and long read sequencing

We extracted bacterial DNA from 1mL of wildtype and phage-resistant variants of *A. baumannii* using the Circulomics© Nanobind high molecular weight genomic DNA extraction kit. We used Oxford Nanopore's Rapid Sequencing kit (RAD004) to produce libraries as per the manufacturer's instructions. We primed a SpotON flow cell (FLO-MIN106D R9) using the flow cell priming kit (EXP-FLP002), and sequenced the library on a MinION Mk1B. Data acquisition was achieved in real time using MinKnow v.# and basecalling was carried out using guppy basecaller (Oxford Nanopore).

In parallel, short read sequencing was carried out by the Exeter sequencing Service. Libraries were prepped using Nebnext library prep and sequenced on the NovaSeq (Illumina).

We used PHASTER (https://phaster.ca/https://phaster.ca/) (Arndt et al., 2016; Zhou et al., 2011) to identify prophage, and manually identified genes involved in capsular polysaccharide (KL) and lipooligisaccharide outer core (OCL) synthesis (Wyres et al., 2020). We used CRISPRCasFinder (Couvin et al., 2018) (https://crisprcas.i2bc.paris-saclay.fr/) and PADLOC (Payne et al., 2022) to detect CRISPRS, spacers and cas genes.

Method S4. Search for evidence of function of hypothetical protein at 2,467,798 bp in the A. baumannii genome

We used the following tools:

InterPro (https://www.ebi.ac.uk/interpro/), HMMER (http://hmmer.org/), JackHammer (https://www.ebi.ac.uk/Tools/hmmer/search/jackhmmer) and NCBI BLASTP (https://blast.ncbi.nlm.nih.gov/Blast.cgi?PAGE=Proteins), String v. 11.5 database and ScanProsite (https://prosite.expasy.org/scanprosite/), CATH (http://www.cathdb.info/search/by_sequence),

CD-Search (https://www.ncbi.nlm.nih.gov/Structure/cdd/wrpsb.cgi), and ColabFold v1.5.2 AlphaFold2 (https://alphafold.ebi.ac.uk/).

Method S5 – Preparation of phage and bacterial inoculums for galleria assays

Bacterial strains and preparation of bacterial inoculum

A. baumannii reference strain NCTC 13420 cells were stored as cryostocks at -80°C, and cultured in LB broth or agar aerobically at 37°C. From streak plates, 20 individual colonies were selected and inoculated into 15mL LB containing $CaCl_2$ and $MgCl_2$ at a final concentration of 10mM, and incubated at 37°C on an orbital shaker at 200rpm for ~2 hours, until OD_{600} = 0.6 (~2 hours). Cultures were centrifuged at 4000rpm for 10 minutes, disposed of the supernatant and re-suspended the pellet in 5ml of 1xPBS. The bacterial suspension was checked and diluted if necessary to OD_{590} = 1 ±0.05. Bacterial colony counts of the inoculum were confirmed on LB agar: mean CFU/mL = 4.8 x 10^8 (ranging from 4.0 to 5.7 x 10^8 CFU/mL across assays) and used this dose in the infection assays.

Preparation and quantification of phage inoculum

Before its use as an inoculum, 1mL phage lysate was re-filtered (0.22nm filter) to ensure no host cells were present. As an additional control, phage solution was spotted out onto a LB agar plate and grown at 37 degrees overnight, to ensure no bacterial cells had contaminated it. We carried out PFU counts and determined the concentration of LemonAid and Tonic to be 5.3 x10^8 and 6.63 X 10^9 PFU/mL respectively. Preliminary experiments revealed that 5.3 x 10^8 PFU/mL phage solution caused melanisation in 50% (N=10) of larvae within 24 hours, compared to no melanisation in control larvae that were inoculated with LB buffer (N=10) or PBS (N=10). No death or malaise resulted from inoculation with 10^7 PFU/mL, thus we used this concentration in all subsequent experiments.

Method S6 – Galleria larvae inoculations and controls

Research-grade *G. mellonella* larvae in their final-instar stage were purchased from Biosystems Technology, TruLarv™. Larvae were stored in the dark at 15°C and used within the week of arrival. The needle was prepared before and between treatments by washing twice in 70% ethanol and twice in 1x PBS; needles were checked for sterility before and after injections by spotting PBS on LB agar, incubating at 37°C overnight and checking for colonies after 24 hours. Separate needles were used for bacteria and phage inoculums.

Method S7 – Endotoxin testing

The endotoxin concentration of the phage preparation was determined using the ToxinSensor chromogenic lyophilised amoebocyte lysate (LAL) endotoxin assay kit (GenScript, New Jersey, USA). The assay was carried out according to the manufacturer's instructions, using one-tenth volumes, in a pyrogen free round bottomed 96 well plate (Grenier Bio-One, Kremsmünster, Austria). Endotoxin standards over the range 0.0 – 1.0 EU/mL were prepared in the LAL reagent water provided. The phage preparation was diluted to 10^{-8} in cell culture grade sodium chloride solution (0.9%, Sigma-Aldrich, St. Louis,

USA). Standards and dilutions of the phage preparation were assayed for endotoxin concentration in triplicate.

Supplementary results

Survival of G. mellonella larvae infected with A. baumannii NCTC-13420 is dose-dependent

Inoculation with *A. baumannii* NCTC-13420 killed larvae in a dose dependent manner (figure S8 below). A 10 µl dose containing 4 x 10^6 cells resulted in 60% (N=10) mortality within 6 hours. There was no killing with an inoculum of 4 x 10^3 cells throughout the 44-hour experiment, in line with PBS control larvae (figure 3a). Killing was dependent on the number of bacterial cells inoculated into larvae (log rank test: $\chi^2 = 45$, 4d.f, $p < 0.001$: pairwise comparison corrected p-values (BH)) showed survival at doses 10^5 and 10^6 cells was significantly different from a dose of 10^3 cells or PBS, $p < 0.001$ for both (table S6). In parallel to the survival data, melanisation was dependent on dose over time (anova: treatment F value = 278.28 on 4d.f; time F value = 36.53 on 8d.f; treatment*time F = 6.03 on 32 d.f; all $p < 0.001$). Larvae inoculated with a dose of 4 x 10^4 - 10^6 cells melanised significantly more than PBS controls and the 4 x 10^3 CFU treatment group, over time (figure S9; table S7).

Figure S9. a) Survival (dashed black lines indicate median survival time per curve) and (b) melanisation (with 95% CI) over time of *G. mellonella* larvae infected with *A. baumannii* strain NCTC 13420 in 1:10 serial dilution, highest dose = 4x 10^6 cells

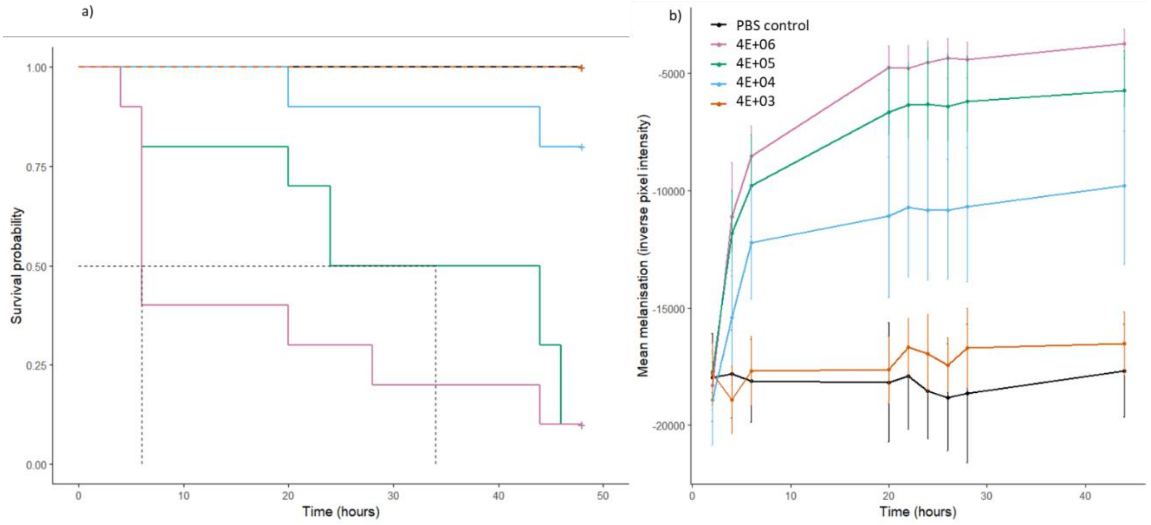

Table S6. Pairwise p-value (adjusted for multiple comparisons using BH method) comparisons of treatments from a survival log-rank test of the dose-response assay.

Dose *A. baumannii*	10^3 cells	10^4 cells	10^5 cells	10^6 cells
**10^4 cells**	0.183	na	na	na
**10^5 cells**	0.000	0.005	na	na
**10^6 cells**	0.000	0.001	0.293	na
**PBS**	1.000	0.183	0.000	0.000

Table S7. Full model of melanisation for dose-response assay, with the negative PBS control as the reference group.

	Estimate	Standard error	t value	p value
Intercept	17955.55	898.65	19.98	0.00
Dose 10^6	357.05	1270.88	0.281	0.78
Dose 10^5	-238.20	1270.88	-0.187	0.85
Dose 10^4	946.0	1270.88	0.744	0.45
Dose 10^3	-139.70	1270.88	-0.110	0.92
Time 4hrs	-145.35	1270.88	-0.114	0.91
Time 6hrs	143.80	1270.88	0.113	0.91
Time 20hrs	213.35	1270.88	0.168	0.86
Time 24hrs	586.45	1270.88	0.461	0.64
Time 26hrs	861.15	1270.88	0.546	0.59
Time 28hrs	694.15	1270.88	-0.22	0.83
Time 44hrs	-279.15	1270.88	-0.22	0.83
**Interactions treatment:time**				
10^6 : 4hrs	-7064.15	1797.29	-3.93	0.00
10^5 : 4hrs	-5764.30	1797.29	-3.20	0.00
10^4 : 4hrs	-3341.95	1797.29	-1.86	0.06
10^3 : 4hrs	1250.50	1797.29	0.70	0.50
10^6 : 6hrs	-9922.80	1797.29	-5.52	0.00
10^5 : 6hrs	-8094.05	1797.29	-4.50	0.00
10^4 : 6hrs	-6833.60	1797.29	-3.80	0.00
10^3 : 6hrs	-272.70	1797.29	-0.15	0.88
10^6 : 20hrs	-13772.40	1797.29	-7.66	0.00
10^5 : 20hrs	-11298.25	1797.29	-6.29	0.00
10^4 : 20hrs	-8043.80	1797.29	-4.48	0.00
10^3 : 20hrs	391.90	1797.29	-0.22	0.83
10^6 : 24hrs	-14379.30	1797.29	-8.00	0.00
10^5 : 24hrs	-11987.45	1797.29	-6.67	0.00
10^4 : 24hrs	-8660.85	1797.29	-4.82	0.00
10^3 : 24hrs	-1461.45	1797.29	-0.81	0.42

References

Arndt, D., Grant, J. R., Marcu, A., Sajed, T., Pon, A., Liang, Y., & Wishart, D. S. (2016). PHASTER: a better, faster version of the PHAST phage search tool. *Nucleic Acids Research*, *44*(W1), W16–W21. https://doi.org/10.1093/nar/gkw387

Couvin, D., Bernheim, A., Toffano-Nioche, C., Touchon, M., Michalik, J., Néron, B., Rocha, E. P. C., Vergnaud, G., Gautheret, D., & Pourcel, C. (2018). CRISPRCasFinder, an update of CRISRFinder, includes a portable version, enhanced performance and integrates search for Cas proteins. *Nucleic Acids Research*, *46*(W1), W246–W251. https://doi.org/10.1093/nar/gky425

Darling, A. C. E., Mau, B., Blattner, F. R., & Perna, N. T. (2004). Mauve: Multiple alignment of conserved genomic sequence with rearrangements. *Genome Research*, *14*(7), 1394–1403. https://doi.org/10.1101/gr.2289704

Katoh, K., & Standley, D. M. (2013). MAFFT multiple sequence alignment software version 7: Improvements in performance and usability. *Molecular Biology and Evolution*, *30*(4), 772–780. https://doi.org/10.1093/molbev/mst010

Kropinski, A., Mazzocco, A., Waddell, T. E., Lingohr, E., & Johnson, R. P. (2009). *Bacteriophages* (M. R. J. . Clokie & A. M. Kropinski, Eds.; Vol. 501). Humana Press. https://doi.org/10.1007/978-1-60327-164-6

Letunic, I., & Bork, P. (2021). Interactive tree of life (iTOL) v5: An online tool for phylogenetic tree display and annotation. *Nucleic Acids Research*, *49*(W1), W293–W296. https://doi.org/10.1093/nar/gkab301

Page, A. J., Cummins, C. A., Hunt, M., Wong, V. K., Reuter, S., Holden, M. T. G., Fookes, M., Falush, D., Keane, J. A., & Parkhill, J. (2015). Roary: Rapid large-scale prokaryote pan genome analysis. *Bioinformatics*, *31*(22), 3691–3693. https://doi.org/10.1093/bioinformatics/btv421

Payne, L. J., Meaden, S., Mestre, M. R., Palmer, C., Toro, N., Fineran, P. C., & Jackson, S. A. (2022). PADLOC: a web server for the identification of antiviral defence systems in microbial genomes. *Nucleic Acids Research*, *50*(W1), W541–W550. https://doi.org/10.1093/nar/gkac400

Wyres, K. L., Cahill, S. M., Holt, K. E., Hall, R. M., & Kenyon, J. J. (2020). Identification of acinetobacter baumannii loci for capsular polysaccharide (KL) and lipooligosaccharide outer core (OCL) synthesis in genome assemblies using curated reference databases compatible with kaptive. *Microbial Genomics*, *6*(3). https://doi.org/10.1099/mgen.0.000339

Zhou, Y., Liang, Y., Lynch, K. H., Dennis, J. J., & Wishart, D. S. (2011). PHAST: A Fast Phage Search Tool. *Nucleic Acids Research*, *39*(SUPPL. 2). https://doi.org/10.1093/nar/gkr485
